# Effect of Simulated Gastrointestinal Digestion on the Phenolic Composition and Bioactivity of *Cymbopogon flexuosus* Extracts

**DOI:** 10.3390/foods14223868

**Published:** 2025-11-12

**Authors:** Ana Alimpić Aradski, Danijel D. Milinčić, Mirjana B. Pešić, Milena Milutinović, Eisuke Kuraya, Akiko Touyama, Danka Bukvički

**Affiliations:** 1 Chair of Morphology and Systematics of Plants, Institute of Botany and Botanical Garden “Jevremovac”, Faculty of Biology, University of Belgrade, Studentski trg 16, 11158 Belgrade, Serbia; 2 Department of Chemistry and Biochemistry, Institute of Food Technology and Biochemistry, Faculty of Agriculture, University of Belgrade, Nemanjina 6, 11080 Belgrade, Serbia; 3 Department for Biology and Ecology, Faculty of Science, University of Kragujevac, Radoja Domanovića 12, 34000 Kragujevac, Serbia; 4 National Institute of Technology, Okinawa College, 905 Henoko, Nago 905-2192, Okinawa, Japan; kuraya@okinawa-ct.ac.jp (E.K.); touyama@flora.okinawa (A.T.)

**Keywords:** *Cymbopogon flexuosus* extracts, in vitro simulated digestion (in vitro GID model), UHPLC Q-ToF MS, phenolic composition, antioxidant activity, antidiabetic activity, cytotoxic activity

## Abstract

This study characterized leaf extracts of *Cymbopogon flexuosus* (Ryukyu Lemongrass Corporation, Okinawa, Japan) and evaluated the bioaccessibility and bioactivities of phenolic compounds following a simulated in vitro gastrointestinal model of digestion (in vitro GID) of plant material. Undigested (controls, AqC, EtC) and digested aqueous (AqD) and ethanolic (EtD) extracts were analyzed. Control extracts contained higher total phenolics and flavonoids than digested ones, with EtC showing the highest values. UHPLC-QToF-MS (ultra-high-performance liquid chromatography system coupled to a quadrupole time-of-flight mass spectrometer) identified 32 compounds, including phenolic acids, flavone aglycones, C-glycosides, and derivatives. Hydroxybenzoic acids, coumaric acid, caffeic esters, flavones, tricin derivatives, vitexin, and isoorientin exhibited reduced recovery, while coumaric acid hexoside, ferulic acid hexoside, and isoschaftoside/schaftoside exceeded 100% recovery, suggesting release from the matrix. Some compounds were absent from AqD, and many were found in the pellet, indicating potential colonic metabolism. Antioxidant activity (DPPH, reducing power, β-carotene/linoleic acid) was stronger in controls but always weaker than BHT/ascorbic acid. Extracts mildly inhibited α-amylase but more strongly inhibited α-glucosidase as shown with applied enzyme inhibition assays, especially EtD (76.93% at a concentration of 10 mg/mL), which showed stronger activity than controls but remained below acarbose (87.74% at 1 mg/mL). All extracts promoted HaCaT keratinocyte growth and reduced HCT-116 colon cancer cell viability at 250 µg/mL, with the strongest effects in AqC and AqD. Overall, GID decreased antioxidant activity but enhanced antidiabetic potential, confirming the safety and selective anticancer effects of *C. flexuosus* extracts.

## 1. Introduction

The genus *Cymbopogon* (lemongrass) belongs to the tribe *Andropogoneae* of the Poaceae family, well known for its high essential oil content. Native to Asia, Africa, Australia, and tropical islands, it comprises 144 species, of which *C. citratus*, *C. flexuosus*, *C. nardus*, and *C. winterianus* are the most commercially relevant. In addition to essential oils rich in citral (a racemic mixture of the isoforms geranial and neral, responsible for the characteristic lemon-like aroma), *Cymbopogon* spp. contains diverse phenolic constituents and flavonoids that support their applications in cosmetics, pharmaceuticals, and phytotherapy [1,2,3,4].

*Cymbopogon flexuosus* (Nees ex Steud) Wats., commonly known as East Indian, Cochin, or Malabar grass, is a tall perennial species that typically grows up to 2 m, forming dense tufts and producing abundant flowers. Two main varieties—red grass and white grass—are distinguished according to stem coloration [5].

*C. flexuosus* is the primary source of lemongrass oil, which may contain up to 90% citral, conferring therapeutic properties and extensive use in aromatherapy and perfumery. Crushed and powdered leaves are consumed in various countries as a culinary ingredient and herbal infusion, traditionally employed to alleviate constipation, coughs, bladder disorders, headaches, fever, flu symptoms, and a wide range of digestive ailments, including stomach pain, diarrhea, flatulence, intestinal cramps, and vomiting. The leaves have also been reported to exert cholesterol-lowering effects [6]. Phytochemical analysis of *C. flexuosus* leaves conducted by Nwauche et al. [7] revealed the presence of phenolic acids, saponins, glycosides, cyanogenic glycosides, alkaloids, anthocyanins, flavonoids, and sterols, underscoring the strong nutraceutical potential of this species.

As reported by Skaria et al. [5], lemongrass and its essential oil exhibit a wide range of biological activities, including analgesic effects in relieving muscle and joint pain, toothache, and headaches associated with viral infections, as well as antidepressant effects linked to mood improvement and attenuation of depressive symptoms. Strong antibacterial and antifungal activities have been demonstrated, particularly against *Candida albicans* and *Aspergillus flavus*. In addition, lemongrass oil exhibits antipyretic, astringent, carminative, diuretic, and deodorizing properties, and it has traditionally been used as a sedative, tonic, and nervine for the treatment of nervous disorders, insomnia, and fatigue.

Research on *C. flexuosus* essential oil has confirmed its antimicrobial, antioxidant, anti-inflammatory, anticancer, chemopreventive, allelopathic, insect-repelling, and anthelmintic activities [2]. Citral, as a major component, has shown strong bactericidal activity against multidrug-resistant *Acinetobacter baumannii* strains, with no cytotoxic effects on human dermal fibroblasts [8]. Furthermore, the oil has been reported to mitigate H_2_O_2_-induced oxidative stress, exhibit photoprotective activity, and exert antiproliferative effects on HepG2 and Calu-1 cancer cell lines [9]. More recently, Júnior et al. [10] demonstrated that a 14-day administration of the essential oil significantly reduced blood glucose levels, improved lipid profiles, and alleviated liver abnormalities in streptozotocin-induced diabetic rats.

By contrast, relatively few studies have addressed the bioactivity of lemongrass extracts. Aqueous leaf extracts of *C. flexuosus* significantly reduced serum glucose levels in diet-induced diabetic mice, probably via α-amylase inhibition or TNF-α suppression [11]. Nomier et al. [12] showed that ethanolic extracts possess antidepressant and anxiolytic effects in rats exposed to chronic mild stress. Ethanolic extracts from the aerial parts of *C. flexuosus* are also rich in phenolic constituents, among which 3-*O*-di-E-caffeoylglycerol and 1-*O*-*p*-coumaroyl-3-*O*-caffeoylglycerol display strong antioxidant and cytotoxic activity against HepG2 and A549 cancer cell lines [13].

Given the potential of lemongrass extracts to mitigate symptoms of prevalent chronic diseases such as diabetes, cancer, and dementia, this species represents a promising candidate for further investigation. However, the richness of its phytoconstituents raises critical questions regarding bioavailability. Numerous studies employing simulated gastrointestinal digestion (GID) models have shown that digestion alters both the content and composition of phenolic compounds, polysaccharides, and tannins [14,15,16,17], leading to the transformation of many components.

Although lemongrass leaves are consumed as tea and their bioactivities have been demonstrated, to our knowledge, there are no published data on the in vitro GID of their leaves, extracts, or essential oils. Previous reports on other plants have shown that simulated GID decreases total phenolic and flavonoid contents as well as the antioxidant activity of extracts [15,16], while simultaneously increasing α-glucosidase inhibition capacity [16]. Therefore, we hypothesize that simulated GID could influence the digestion of lemongrass leaves in a similar manner.

Accordingly, the objective of the present study was to characterize *C. flexuosus* leaf extracts and to evaluate the bioaccessibility and recovery of phenolic compounds, as well as their antioxidant, antidiabetic, and cytotoxic properties, following the GID protocol.

## 2. Materials and Methods

### 2.1. Chemicals and Reagents

Acarbose, ascorbic acid, BHT (3,5-di-tert-butyl-4-hydroxytoluene), DPPH (2,2-diphenyl-1-picrylhydrazyl), β-carotene, bile salts iron(III) chloride, potassium chloride, magnesium chloride, Lugol’s solution, Folin–Ciocalteu reagent, potassium acetate, aluminium nitrate nonahydrate, pNPG (4-nitrophenyl β-D-glucopyranoside), potassium dihydrogen phosphate, potassium hydrogen phosphate, sodium bicarbonate, sodium carbonate, sodium chloride, sodium dihydrogen phosphate, sodium hydrogen phosphate, and calcium carbonate were purchased from Sigma-Aldrich (St. Louis, MO, USA). Linoleic acid and Tween 40 were obtained from Acros Organics (Geel, Belgium). Starch solution (1%) was purchased from Carl Roth (Karlsruhe, Germany). Potassium ferricyanide(III), trichloroacetic acid, and ethanol were obtained from Superlab (Belgrade, Serbia), while chloroform and hydrochloric acid were obtained from Zorka Pharma (Šabac, Serbia). All reagents were of analytical purity. LC-MS grade methanol, acetonitrile, and formic acid were obtained from Carlo Erba (Val-de-Reuil, France), while ultrapure water was prepared using a purification system BBPS-508 (Biolab Scientific, Toronto, Canada). Alpha-amylase from porcine pancreas (type VI-B, ≥5 units/mg solid), α-glucosidase from *Saccharomyces cerevisiae* (type I, ≥10 units/mg protein), α-amylase from human saliva (type IX-A, 1000–3000 units/mg protein). Pancreatin from porcine pancreas and pepsin from pig gastric mucosa (≥250 units/mg protein) were purchased from Sigma-Aldrich, USA.

### 2.2. Plant Material

**Figure 1:**
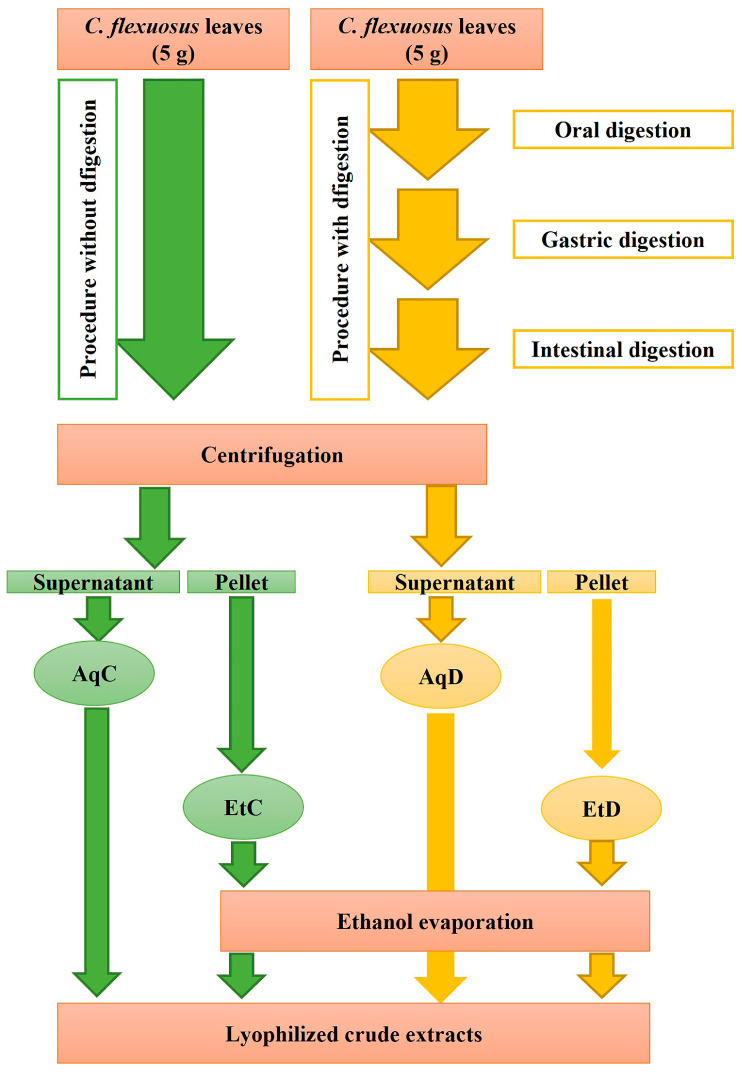
The experimental procedure applied in order to obtain ethanolic (Et) and aqueous (Aq) digested (D) and control (C) extracts. The digested extracts were exposed to the stimulated enzymatic digestion through steps of oral, gastric, and intestinal digestion, while control samples were prepared without enzymatic digestion.

*Cymbopogon flexuosus* was provided by the Ryukyu Lemongrass Corporation (Nago City, Okinawa, Japan) in July 2024. The plant species was identified by Dr. Eisuke Kuraya, and the voucher specimens were deposited at MAK (A. Touyama # CYFL 010). Five grams of dried fresh leaves are used for simulated gastrointestinal digestion to prepare digested samples, and 5 g are used to prepare the control samples (Figure 1).

### 2.3. In Vitro Simulated Gastrointestinal Digestion

In vitro simulated GID of *C. flexuosus* leaves was performed using the protocol and terminology previously described by Diab et al. [15] with slight modifications. To simulate digestion in the mouth, simulated salivatory fluid (SSF) was prepared as shown in Table 1, followed by stomach (simulated gastric fluid—SGF) and small intestine (simulated intestinal fluid—SIF).

Oral digestion: To mimic oral digestion, 5 g of air-dried and milled leaves of *C. flexuosus* were mixed with 5 mL of SSF and 0.6 mL of α-amylase from human saliva (stock 75 U/mL), 0.04 mL of calcium carbonate, and 1.14 mL of distilled water. The mixture was incubated for 2 min at 37 °C on the magnetic stirrer.

Gastric digestion: To mimic gastric digestion, 8 mL SGF, 1.4 mL pepsin (stock 25,000 U/mL), 0.006 mL calcium carbonate, and 0.6 mL distilled water were added to the oral outcome. Then the pH of the mixture was lowered to 3.0 with hydrochloric acid, and incubation was continued for 2 h at 37 °C on the magnetic stirrer.

Intestinal digestion: To mimic intestinal digestion, 10 mL of gastric outcome was mixed with 10 mL SIF, 4 mL pancreatin (stock 100 U/mL), 2 mL bile salts (stock 10 mM), 0.0048 mL calcium carbonate, 1.2 mL distilled water and 0.14 mL 1 M hydrochloric acid to neutralize the pH to 7.0, and incubation was continued for 2 h at 37 °C on the magnetic stirrer.

Preparation of digested and control samples for analysis: To inactivate the enzymes used in the digestion, the mixture was heated to 90 °C for 10 min. After cooling, the samples were centrifuged at 4000 rpm for 20 min, and the supernatant and solid pellet of digested leaves were separated. The supernatant was immediately lyophilized to obtain the crude digested aqueous extract (AqD). The solid pellet of digested leaves was extracted with 96% ethanol for 24 h at room temperature. After centrifugation (20 min, at 4000 rpm), the ethanolic extract was evaporated using a rotary evaporator to remove ethanol, and the remaining residue was lyophilized to yield the crude digested ethanolic extract (EtD), containing compounds retained in the pellet. These originated from the solid pellet of digested leaves, may potentially be available in the colonic phase. The undigested (control) ethanolic (EtC) and aqueous (AqC) extracts were prepared using the same procedure, except that enzymatic digestion step was omitted. All steps of the digestion and extraction procedure are illustrated schematically (Figure 1).

### 2.4. The Determination Total Phenolic (TPC) and Flavonoid (TFC) Content

The contents of total phenolics (TPC) and total flavonoids (TFC) in the digested and control extracts of *C. flexuosus* were determined according to the methods developed by Singleton and Rossi [18] and Park et al. [19], respectively. For the quantification of TPC and TFC, the crude extracts were dissolved in the corresponding solvent to reach a concentration of 5 mg/mL. The spectrophotometric measurements were recorded using the Multiskan Sky Thermo Scientific (Finland) at 740 and 415 nm, respectively. The results of TPC are expressed as gallic acid equivalents per gram of dry extract (mg GAE/g) and those of TFC as quercetin equivalents per gram of dry extract (mg QE/g).

### 2.5. UHPLC Q-ToF MS Analysis and Bioaccessibility

Before analysis, lyophilized extracts (10 mg) were reconstituted in 1 mL of 80% methanol, vigorously vortexed, and additionally mixed on a mechanical shaker for 1 h. After that, samples were centrifuged (17,000× *g*, 5 min), filtered through 0.22 µm filter, and used for chromatographic analysis. Separation, identification and characterization of phenolic compounds in control and digested *C. flexuosus* extracts/samples were performed on an Agilent 1290 Infinity ultra-high-performance liquid chromatography (UHPLC) system coupled to a quadrupole time-of flight mass spectrometer (6530C Q-ToF-MS) (Agilent Technologies, Inc., Stevens Creek Blvd, Santa Clara, CA, USA), using the same column (Zorbax C18 column; Agilent Technologies, Inc., CA, USA), and previously established and detailed described UHPLC method (injection volume, flow rate, and applied gradient elution program) and ESI (Dual Agilent Jet Stream electrospray ionization source) operating parameters [20]. Spectra were recorded in Auto MS/MS acquisition mode using the same parameters (*m*/*z* = 100–1700 and 1 spectra/s scan rate), in both positive and negative ionization modes (ESI^+^/ESI^−^). Data-dependent acquisition (DDA) was employed for suspect screening using the AutoMS/MS acquisition mode. Agilent MassHunter software (AgilentMassHunter Qualitative Analysis 10.0), combined with MS-DIAL (ver. 4.60) software (http://prime.psc.riken.jp/), was used for screening, collection data of interest (retention time, monoisotopic mass, area), evaluation, analysis, and presentation of MS data. Phenolic compounds were tentatively identified based on their monoisotopic mass (serving to predict formula) and typical MS fragments, and additionally confirmed by comparison with available standards and/or literature data [21,22,23,24,25,26,27,28,29]. To further clarify the identification of phenolic compounds, the MS/MS fragmentation patterns (MS/MS spectra) for all identified compounds were exported from AgilentMassHunter software (AgilentMassHunter Qualitative Analysis 10.0) and are provided in the Supplementary Material (Figures S1 and S2). Accurate masses of identified components and fragment ions were calculated using ChemDraw software (version 12.0, CambridgeSoft, Cambridge, MA, USA). The CAS SciFinder-n (https://scifindern.cas.org/) and PubChem (https://pubchem.ncbi.nlm.nih.gov/) databases were applied to search formulas and structures of identified compounds.

Peak areas before and after in vitro gastrointestinal digestion were used to evaluate the recovery (bioaccessibility) of each identified phenolic compound. The peak areas can be reliably compared between undigested and digested samples, as all were processed under identical conditions: the same amount of lyophilized extract was reconstituted in an equal volume of solvent, with identical injection volumes and chromatographic settings. Furthermore, normalized peak areas before and after GID for each compound were processed under the same conditions and exported from MS DIAL software.

The bioaccessibility (recovery) (%) and sediment retained (%) for all identified phenolic compounds (BCs) were calculated using the following Equations:

$$\mathrm{Recovery}\;1(R1,\;\%)=\;\frac{PC\;AqD}{PC\;AqC}\;\times \;100$$

$$\mathrm{Recovery}\;2\;(R2,\;\%)=\;\frac{PC\;AqD}{PC\;EtC}\times 100$$

$$\mathrm{Pellet}\;\mathrm{retained}\;\mathrm{compound}\;(\mathrm{PRC},\;\%)=\frac{PC\;EtD}{PC\;EtC}\times 100$$

where PC AqD is the peak area of each identified compound in the aqueous digested extract; PC AqC is the peak area of each identified compound in the aqueous control extract; PC EtD is the peak area of each identified compound in ethanolic extract of pellet obtained after simulated digestion (bioactive compound retained in the solid residue of digested leaves); PC EtC is the peak area of each identified compound in the ethanolic control extract. Compounds contained in the crude digested ethanolic extract were derived from the solid pellet remaining after in vitro gastrointestinal digestion. Recovery 1 (R1, %) represents the actual recovery of each identified compound following simulated digestion; Recovery 2 (R2, %) reflects recovery relative to maximum potential extractability, assuming that ethanol enables high extraction efficiency of *C. flexuosus* bioactives. There is no single extraction solvent capable of ensuring maximum recovery of all compounds from *C. flexuosus* leaves, as this plant contains phenolic compounds with diverse polarities. However, ethanol is an effective solvent that has been widely applied for extracting a broad range of phenolics. Considering this, ethanol was selected as the extraction agent due to its polarity, which allows efficient extraction (“maximum extractability”) of both highly and moderately polar phenolic compounds. Nevertheless, it should be noted that the assumption of “maximum extractability” may introduce a minor bias in R2 values, as 96% ethanol does not equally extract highly polar glycosides compared with less polar phenolics. Pellet retained compound (PRC, %) indicates the proportion of each compound retained in the pellet after in vitro gastrointestinal digestion, potentially available for further microbial metabolism in the colon.

### 2.6. Biological Activity of Control and Digested Samples

For the evaluation of the antioxidant and antidiabetic activity of the digested and control ethanolic and aqueous extracts of *C. flexuosus*, a double dilution series of 7 concentrations (0.15625, 0.3125, 0.625, 1.25, 2.5, 5, and 10 mg/mL) was prepared, using appropriate solvents. The dilutions of the extracts were colored so that the absorbance of the reaction mixture (As) for all assays was corrected by a color control (CC) containing only the extract and the appropriate solvent (As-CC). All original experimental protocols for biological activity assays were modified to adjust the aliquots for the microtiter plates, and absorbance values were recorded using the Multiskan Sky Thermo Scientific Microtiter plate reader (Vantaa, Finland).

#### 2.6.1. Antioxidant Assays

Three parallel assays (DPPH, Total Reducing Power (TRP), and β-carotene bleaching assay) were performed to evaluate the antioxidant activity of control and digested extracts of *C. flexuosus*. A synthetic antioxidant (BHT) and a natural antioxidant (ascorbic acid, AA) were used as positive controls.

DPPH assay: The free radical scavenging activity of the digested and control ethanolic and aqueous extracts of *C. flexuosus* was evaluated using the most commonly used DPPH assay [30] with slight modifications. The aliquots of the different concentrations of *C. flexuosus* extracts or positive controls with a concentration of 1 mg/mL (20 µL) and 180 µL of a freshly prepared methanolic DPPH solution (40 µg/mL) were mixed. The negative control contained methanol instead of the extracts/standards. After 30 min of incubation in the dark at room temperature, the absorbances were measured at 517 nm. The inhibition rate of DPPH radicals at 517 nm was calculated as follows:

Inhibition of DPPH radical (%) = [(Ac − As)/Ac] × 100,

where Ac represents the absorbance of the negative control and As represents the absorbance of the tested samples at different concentrations.

Total Reducing Power (TRP) assay: The Fe(III) reducing power of the samples was evaluated according to Oyaizu method, with the modifications described by Tusevski et al. [31]. In brief, 20 µL of the different concentrations of control and digested *C. flexuosus* extracts or positive controls at a concentration of 1 mg/mL were mixed with 0.2 M phosphate buffer (40 µL, pH 6.6) and 1% potassium ferricyanide (III) solution (40 µL). After incubation (20 min, 45 °C), 10% trichloroacetic acid (40 µL), distilled water (40 µL), and 0.1% iron (III) chloride (8 µL) were added. After incubation for 10 min at 45 °C, the absorbances of the reaction mixtures were measured at 700 nm. The negative control was prepared to contain distilled water instead of the sample, while dilutions of ascorbic acid (AA) (7.81–2000 µmol/L) were used to prepare the calibration curve. The TRP values of the samples were expressed as µM ascorbic acid equivalents (AAE) per gram of dry extract (µM AAE/g).

β-Carotene bleaching assay: The control and digested *C. flexuosus* extracts were tested for their ability to inhibit oxidative bleaching of β-carotene in the β-carotene/linoleic acid system. This assay was performed as described by Dapkevicius et al. [32], with slight modifications. The emulsion was prepared by adding linoleic acid (6.25 µL) and Tween 40 (50 mg) to a solution of β-carotene in chloroform (250 µL, 2 mg/mL). The chloroform was then removed using a rotary evaporator (Buchi rotavapor R-114, Marshall Scientific, Hampton, NH, USA) at 40 °C, and distilled water (25 mL) was added, and the emulsion was shaken vigorously. Aliquots of 28 µL of the *C. flexuosus* extracts at various concentrations and positive controls at concentrations of 1 mg/mL were mixed with the previously prepared emulsion (200 µL). The negative control contained distilled water instead of a sample. The absorbance was measured twice, immediately after mixing the sample and emulsion, and after 120 min of incubation at 490 nm. The antioxidant activity of the samples was evaluated as inhibition of β-carotene bleaching as follows:

Inhibition of β-carotene bleaching (%) = [(A120 − C120)/(C0 − C120)] × 100,

where A120 and C120 are the absorbance values of the sample and negative control after 120 min, respectively, while C0 is the absorbance value of the negative control measured immediately after mixing the sample and emulsion.

#### 2.6.2. Antidiabetic Assays

The antidiabetic activity was investigated using α-glucosidase and α-amylase inhibition assays. The commercial inhibitor for both enzymes and the commonly used antidiabetic drug acarbose were used as a positive control.

α-Amylase inhibition assay: The inhibition of α-amylase was evaluated using the slightly modified Caraway–Somogyi iodine/potassium iodide method according to the experimental protocol described in detail by Zengin et al. [33]. The aliquots (25 µL) of control and digested *C. flexuosus* extracts, properly diluted with appropriate solvents at different concentrations, and acarbose at a concentration of 1 mg/mL, were mixed with 50 µL of α-amylase solution (0.5 mg/mL) prepared with sodium phosphate buffer (0.1 M, pH 6.8 with 6 mM sodium chloride). The reaction mixture was pre-incubated (10 min, 37 °C) prior to the addition of 0.2% starch dissolved in phosphate buffer (50 µL). The reaction mixture was then incubated (10 min, 37 °C). The reaction was stopped by adding 1 M hydrochloric acid (25 µL) and finally visualized by mixing with Lugol’s solution (100 µL). The absorbance of the reaction mixture was measured at 630 nm. The inhibition of α-amylase activity was calculated according to the following equation:

Inhibition of α-amylase (%) = [(As − Ac1)/Ac2] × 100,

where As is the absorbance of the reaction mixture with the test sample, Ac1 is the absorbance of the enzyme control (with buffer instead of the sample), and Ac2 is the absorbance of the substrate control (with buffer instead of the enzyme).

α-Glucosidase inhibition assay: The ability of control and digested *C. flexuosus* extracts to inhibit α-glucosidase activity was performed as described by Wan et al. [34]. Aliquots (120 µL) of the extracts at different concentrations and acarbose were mixed with an enzyme solution (20 µL, 0.5 units/mL) prepared in potassium phosphate buffer (0.1 M, pH 6.8). After pre-incubation (5 min, 37 °C), 5 mM pNPG (20 µL) was added, and the mixture was additionally incubated (20 min, 37 °C). The reaction was terminated by adding 0.2 M anhydrous sodium carbonate dissolved in a buffer (80 µL), and the absorbance was measured at 405 nm. The percentage of inhibition of α-glucosidase activity was calculated as follows:

Inhibition of α-glucosidase (%) = [(Ac − As)/Ac] × 100,

where Ac represents the absorbance of the negative control (contained buffer instead of the sample) and As represents the absorbance of the reaction mixture with the test sample.

#### 2.6.3. Cytotoxicity Assay

Cell culturing: The normal human HaCaT keratinocytes and the HCT-116 colorectal carcinoma cell line, cultured according to the standard protocols described in detail in Milutinović et al. [35], were purchased from the CLS-Cell Lines Service (Eppelheim, Germany) and the American Tissue Culture Collection (Manassas, VA, USA), respectively.

Evaluation of Cytotoxicity: To quantify the viability of normal (healthy) and cancer cells and to determine the cytotoxicity of control and digested *C. flexuosus* extracts, the MTT (3-(4,5-dimethylthiazol-2-yl)-2,5-diphenyltetrazolium bromide) assay was used [36]. The viable cells metabolize the yellow MTT by reducing it to violet formazan, which can be detected spectrophotometrically. For this experiment, 10^4^ cells per well were incubated for 24 h in a 96-well plate to adhere. The treatments were applied for each extract in a concentration range of 10–250 µg/mL, diluted in 100 µL culture medium. Cells in the culture medium only, without the treatment, were used as a control. After 24 and 72 h of incubation, the dissolved formazan (in 150 µL DMSO) was measured. The observed absorbance values are directly proportional to the number of metabolically active cells, which is an indirect measure of cell viability. The IC50 values were calculated in CalcuSyn, and Emax with 95% CI were observed in GraphPad Prism (version 10.6.1).

### 2.7. Statistical Analysis

With the exception of the digestion and chemical analyses, all measurements were performed in triplicate and expressed as mean values ± standard deviation. The significance of the differences between the mean values was tested using PAST (PAleontological STatistics) 4.17 by analysis of variance (one-way ANOVA) and Tukey’s post hoc test [37].

## 3. Results and Discussion

### 3.1. Total Phenolic (TPC) and Flavonoid (TFC) Content

**Figure 2:**
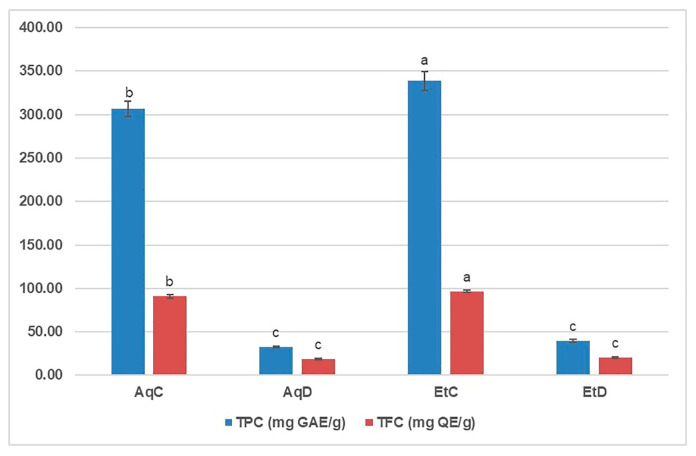
Total phenolic content (TPC) and total flavonoid content (TFC) of digested (D) and control (C) aqueous (Aq) and ethanolic (Et) extracts of *C. flexuosus* at concentration of 5 mg/mL. Mean values with different superscript letters (a–c) show significantly different TPC or TFC (one-way ANOVA, Tukey’s post hoc; *p* < 0.05). The superscript letters indicate the values in descending order, with (a) representing the highest value.

Although many studies have shown that phenolic compounds from edible and/or medicinal plants have beneficial effects on human health, few reports have investigated the possible influence of gastrointestinal digestion on their efficacy [15]. The total phenolic and total flavonoid content (TPC and TFC, respectively) of the digested and control *C. flexuosus* extracts were determined spectrophotometrically at a concentration of 5 mg/mL (Figure 2).

As the results showed, the TPC values of the undigested (control) extracts were about 8.5–9.5 times higher than those of the digested extracts, while this ratio in the case of TFC was 4.7–4.9 (Table 6). The EtC had significantly higher TPC and TFC values than AqC (*p* < 0.05), while the differences between the corresponding digested extracts were not significantly different. Le et al. [13] also obtained higher values of TPC and TFC for 70% ethanol than for hot water fraction, both obtained from the total extract of *C. flexuousus* aerial parts. Similarly, Diab et al. [15] found a higher TPC value in the ethanolic extract of *Thymbra spicata* than in the aqueous extract, while the TFC values were higher in the aqueous extract than in the ethanolic extract. In both cases, simulated digestion led to a 1.4 to 1.6-fold decrease in TPC and TFC values, whereas in the present study, the decrease was more pronounced. Ma et al. [16] observed the decrease in TPC and TFC during simulated in vitro digestion of winter jujube fruit and hypothesized that digestive enzymes and pH may contribute to the degradation or transformation of phenolic compounds, ultimately leading to a decrease in the amount of total phenolics and flavonoids. At the end of the intestinal phase, i.e., after pancreatic digestion, the drastic reduction in polysaccharide content was also observed [17], as well as in punicalin and punicalagin from the pomegranate extract, while the ellagic acid content was increased [14].

### 3.2. UHPLC Q-ToF MS Identification and Characterization of Phenolic Compounds in Control and Digested Extracts

Comprehensive UHPLC Q-ToF MS analysis revealed 32 compounds in aqueous and ethanolic extracts before and after in vitro digestion of *C. flexuosus* leaves, as presented in Table 2. All identified compounds belonged to (a) phenolic acids (hydroxybenzoic acid and hydroxycinnamic acid derivatives); (b) flavonoids (flavone aglycones, flavone O-glycosides, flavone C-glycosides, and flavonolignans); and (c) another compound (quinic acid). The MS base peak chromatograms of *C. flexuosus* extracts, in negative and positive ionization modes, are presented in Figure S4. Peaks of the identified compounds were extracted from the chromatographs. Most of the identified compounds correspond to phenolic acid derivatives, flavone aglycones, and glycosides (*O*- and *C*-glycosides), for which no authentic standards are commercially available. Among the detected phenolic compounds, only a few were confirmed by direct comparison with available standards (Category 1#-confirmed compounds; these compounds are marked in Table 2 of the revised manuscript). Other phenolic compounds were identified based on their accurate *m*/*z* mass (monoisotopic mass), used to predict molecular formulas and their characteristic MS/MS fragments (Category #2-tentatively identified compounds and were further confirmed by comparison with literature data. Representative references reporting these compounds in various *Cymbopogan* species are listed in Table 2.

**Figure 3:**
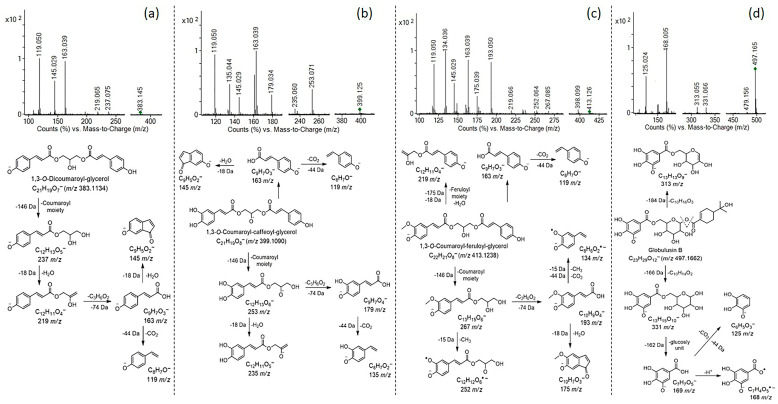
Characteristic MS/MS fragmentation patterns (collision-induced dissociation (CID) mass spectra) and proposed fragmentation pathways of: (**a**) 1,3-*O*-Dicoumaroyl-glycerol (*m*/*z* 383); (**b**) 1,3-*O*-Coumaroyl-caffeoyl-glycerol (*m*/*z* 399); (**c**) 1,3-*O*-Coumaroyl-feruloyl-glycerol (*m*/*z* 413); (**d**) Globulusin B (*m*/*z* 497); (Agilent, Q-ToF, ESI(–), CE = 30 eV). All fragment ions from MS/MS fragmentation patterns are included, explained, and annotated in the proposed fragmentation pathways.

Phenolic acids were mostly confirmed in the analyzed extracts in negative ionization mode, in the form of monoglycosides, esters with quinic acid, or derivatives with glycerol. Among glycosides, coumaric acid hexoside (**10**), ferulic acid hexoside (**11**), and two isomers of dihydroxybenzoic acid hexoside (**4** and **5**) were confirmed, considering unique monoisotopic mass, retention time differences, and typical fragments obtained by the loss of hexosyl unit (−162 Da) and CO_2_ group from phenolic acid aglycones (−44 Da). Coumaroylquinic acid (337 *m*/*z*; **12**), isomers of caffeoylquinic acid (353 *m*/*z*; **13**–**14**) and dicaffeoylquinic acid (515 *m*/*z*; **15**) were identified based on typical fragments obtained by the loss and cleavage of (a) deprotonated quinic acid [191→173(-H_2_O)→155(-H_2_O)→111(-CO_2_) *m*/*z*]; and (b) coumaric acid aglycone [163→119 (-CO_2_) *m*/*z*] or caffeoyl aglycone [179→161(-H_2_O) *m*/*z*; 135 (-CO_2_) *m*/*z*]. Compounds **17**, **18**, and **19** were recognized as 1,3-*O*-phenolic acids (coumaric, caffeic and/or ferulic acid)-glycerol derivatives with well-known: (a) fragments corresponding to phenolic acid aglycones; and (b) fragments formed by losses of coumaroyl (−145 Da), caffeoyl (−162 Da) and/or feruloyl (−175 Da) moiety(es) from precursor ions. Characteristic MS/MS fragmentation patterns, proposed structures, and fragmentation pathways of the detected phenolic acids-glycerol derivatives are presented in Figure 3a–c. In addition to the aforementioned phenolic acid derivatives, ethyl caffeate (**8**) and globulusin B (**6**) were also confirmed. Globulusin B was previously reported in *Eucalyptus globulus* leaves [23], and it was identified based on typical fragments obtained by sequential losses of oleuropeic acid and glucosyl unit to galloyl moiety [169/168 *m*/*z*→125/124 (-CO_2_) *m*/*z*], as shown in Figure 3d. All labeled fragment ions in the presented MS/MS fragmentation patterns of phenolic acid-glycerol derivatives and globulsin B are included, explained, and annotated in the proposed fragmentation pathways (Figure 3a–d). Phenolic acids, such as hydroxybenzoic (**1**), protocatechuic (**2**), gentisic (**3**), coumaric (**7**), and caffeic (**8**) acid were also detected in the analyzed extracts. Mentioned phenolic acids were further confirmed using available standards, except for hydroxybenzoic acid. Phenolic acids, some cinnamate esters, and phenolic acid-glycerol derivatives have also been previously reported in *Cymbopogon citratus* extracts [21,25,26,27,38].

All identified flavonoids in *C. flexuosus* extracts belonged to the flavone group, and include various aglycones, O-glycosides, C-glycosides, and flavonolignans. Luteolin and tricin were the only aglycones detected in the extracts. Luteolin was detected based on typical fragments at 133 *m*/*z* [^1,3^B^−^], 151 *m*/*z* [^1,3^A^−^], and 107 *m*/*z* [^0,4^A^−^], produced by retro-Diels–Alder (RDA) reaction, and additionally confirmed by comparison with an available standard. Tricin is methylated flavone (3′,5’-di-O-methyltricetin) with main fragments at 314 and 299 *m*/*z*, derived from sequential losses of two methyl groups [*m*/*z* 329→314(-CH_3_) *m*/*z* →299(-CH_3_) *m*/*z*]. Tricin was also detected in the form of glycosides and lignan-derivatives (tricin-lignans). Compound **22** (*m*/*z* 493) was recorded in positive ionization mode, with the main fragment at 331 *m*/*z*, resulting from the loss of a hexosyl unit in the C−_7_ or C−_5_ position, which corresponds to Tricin 7-*O*-hexoside or Tricin 5-*O*-hexoside. Compounds **23** and **24** were identified as tricin-lignan and (tricin-glucoside)-lignan, respectively; both were previously reported in *C. citratus* leaves [21], and in cereals such as rice, barley, maize, wheat, etc. [22]. MS/MS fragmentation of the deprotonated molecular ion at *m*/*z* 525 (**25**) showed fragments at 329 *m*/*z*, 314 *m*/*z*, and 299 *m*/*z*, resulting from sequential losses of guaiacylglyceryl moiety (−196 Da), and two methyl groups from tricin aglycone, corresponding to Tricin 4′-*O*-(*erythro*-*β*-guaiacyl-glyceryl)ether. The proposed structure was additionally supported by a high intensity fragment at 165 *m*/*z*, generated by losses of H_2_O and CH_3_ from the guaiacylglyceryl moiety. Compound **24** was identified as Tricin 4′-*O*-(*erythro*-*β*-guaiacyl-glyceryl)ether-7-*O*-hexoside (*m*/*z* 687), with the same MS/MS fragments as previously reported for compound **23**, and an additional fragment at 525 *m*/*z* showing loss of the hexosyl unit in the C-_7_ position.

Flavone-6-*C*-glycosides were recorded in positive ionization mode, with typical fragments obtained by cross-ring cleavage of the sugar unit(s), and neutral loss(es) of H_2_O molecule(s), as reported by Colombo et al. [39]. Compounds **25** (*m*/*z* 433), **27** (*m*/*z* 449), and **30** (*m*/*z* 463) were recognized as isovitexin, isoorientin, and isoscoparin, respectively. The key fragments for identification of these compounds were obtained by ^0.1^X_6_^+^ scission ([M+H-150]^+^), ^0.2^X_6_^+^ scission ([M+H-120]^+^), and ^0.4^X_6_^+^ scission-2H_2_O ([M+H-96]^+^) of the glucosyl unit. Isovitexin was further confirmed by comparison with an available standard. Compounds **26** and **28** were identified as apigenin-6,8-*C*-pentoside hexoside (Isoschaftoside or Schaftoside; *m*/*z* 565) and luteolin-6,8-*C*-pentoside hexoside (Isocarlinoside or Carlinoside; *m*/*z* 581), respectively. Both exhibited similar MS/MS fragmentation patterns, with fragments derived from cross-ring cleavage of pentosyl (^0.5^Z^+^ to ^0.8^Z^+^, pentose fragmentation) and hexosyl (^0.1^Z^+^ to ^0.4^Z^+^, hexose fragmentation) units: ^0.1^Z^+^+2H_2_O ([M+H-186]^+^); ^0.6^Z^+^+^0.1^Z^+^ or ^0.5^Z^+^+^0.2^Z^+^ ([M+H-240]^+^); ^0.5^Z^+^+^0.1^Z^+^ ([M+H-270]^+^); ^0.5^Z^+^+2H_2_O ([M+H-156]^+^); ^0.2^Z^+^ ([M+H-120]^+^); ^0.6^Z^+^ ([M+H-90]^+^). Identified flavone-C-glycoside(s) were previously found and reported in *C. citratus* leaf extract [21] and/or infusion [25,26].

Compound **29** was recognized as Isoorientin 4′-*O*-glucoside (*m*/*z* 611), with main fragments at 329 *m*/*z* ([M-hexosyl unit-^0.2^X_6_^+^]) and 449 *m*/*z* (loss of *O*-linked glucosyl unit; −162 Da). Compound **31** was recorded in negative ionization mode and identified as acacetin-6-*C*-(6″-*O*-malonyl)glucoside, and this compound was previously reported in the acetone extract of *Aquilegia vulgaris* [24]. Finally, in addition to phenolic compounds, free quinic acid (**32**) was also found in the analyzed extract.

The detected phenolic acids (such as *p*-coumaric acid derivatives) originate from the phenylpropanoid pathway [40], while the identified flavone aglycones and glycosides belong to the flavonoid branch of this biosynthetic route [41]. These metabolites are synthesized via stepwise hydroxylation and O-/C-glycosylation reactions, catalyzed by phenylalanine ammonia-lyase (PAL), chalcone synthase (CHS), and specific glycosyltransferases, which are characteristic of higher plant species [42].

### 3.3. Bioaccessibility of Phenolic Compounds After In Vitro GID of C. flexuosus Leaves

Actual (R1, %) and relative (R2, %) recovery of individual bioactive compounds of *C. flexuosus* leaves, as well as the proportion of phenolic compounds retained in pellet (PRC, %), after in vitro gastrointestinal digestion, are shown in Table 3. Most identified compounds were detected in both aqueous and ethanolic control extracts, as well as in the digested extracts, although they exhibited varying stability and recovery levels (actual and relative recovery) after gastrointestinal digestion. Actual recovery (R1) provides more realistic values, compared to relative recovery (R2), which is based on the “maximal” (high) extractability of most phenolic compounds from *C. flexuosus* leaves. As shown, most compounds displayed low recovery or were complete absent in digesta, indicating poor stability during digestion. In case of applied in vitro GID model (included oral, gastric, and intestinal phases), recovery of phenolic compounds largely depends on the composition of the digestive cocktail as well as on physicochemical parameters (pH, temperature, solubility, polarity), and the nature of plant material itself. Each of the digestive phases has a distinct composition of digestive cocktail and physicochemical environment that can influence the solubility and stability of phenolic compounds, as well as their extraction from the plant matrix. In addition, each plant material possesses unique characteristics and the ability to release bioactive compounds in digestive conditions during digestion. For this digestion model, transformations of phenolic conjugates are considered less likely. The presence or absence of specific phenolic compounds in mixed micelles mainly depends on their solubility, stability within the digestive cocktail, interaction with enzymes, and the extraction capacity of the digestive fluids [43,44], rather than the transformation of phenolic conjugates. Individually, detected hydroxybenzoic acids (**1–3**), glycosides (**4** and **5**), and derivatives (**6**) showed significantly reduced recovery (<16.07%) and pellet retention (<26.50%). However, these compounds had similar actual and relative recovery, except for hydroxybenzoic acid, with higher actual recovery. Coumaric acid showed low recovery, while caffeic acid and ethyl caffeate were not found in aqueous digested extract (AqD, 0%). In contrast, coumaric acid hexoside exhibited recovery rates exceeding 100%, while ferulic acid hexoside was only found in AqD, suggesting release of these glycosides from the leaf matrix during in vitro GID. Coumaric acid and both glycosides were also present in the pellet in significantly higher proportions than in the ethanolic control extract (EtC), making them potentially available in the colon phase. Coumaroylquinic acid showed reduced recovery (46.05% and 63.24%) and pellet retention (43.81%). Caffeoylquinic acid isomers I and II were not detected in aqueous digested extract (AqD), chlorogenic acid showed very low recovery (<1%), while dicaffeoylquinic acid had significantly reduced recovery. These caffeoylquinic derivatives (**13**–**16**) were totally absent or present only in traces in the pellet. Coumaric, caffeic, and/or ferulic acid-glycerol derivatives (**17**–**19**) were not detected in aqueous control (AcC) and digested (AqD) extracts, showing no recovery (0%), as well as poor pellet retention (0–5%). The absence or reduced recovery of phenolic acids, cinnamate esters, and glycerol derivatives may be due to their degradation, ability to form phenolic acid derivatives through polymerization and autooxidation reactions, and reduced stability in GID conditions.

Flavone aglycones (**20** and **21**) showed low active and relative recovery, as well as scarce presence in the pellet. Tricin-lignan (**23**) was not detected in the aqueous digested extract (AqD), but appeared only in the control extracts, indicating absent recovery after in vitro GID. In contrast, tricin-hexoside (**22**) and (tricin-glucoside)-lignan (**24**) showed high recovery, particularly actual recovery (68.81% and 86.91%), together with a high proportion in EtD (compounds retained in pellet) (77.38% and 120.56%), probably due to increased polarity of these glycosylated compounds [45]. In contrast, most of the identified flavone-*C*-glycosides were not detected in the aqueous digested extract (**28**–**31**) or showed very low recovery (<3%) (**25** and **27**), with the exception of Isoschaftoside/schaftoside (**26**). Similarly to our results, Cattivelli et al. [46] reported good stability and bioaccessibility of flavone-*O*-glycosides after digestion of selected vegetables and teas, as well as significantly reduced recovery of some flavone-*C*-glycosides. Isoschaftoside/schaftoside exhibited recovery rates exceeding 100%, suggesting that it may be released from the leaf matrix during in vitro GID. Moreover, Shen et al. [47] also reported a high retention rate and bioaccessibility of isoschaftoside and schaftoside after the digestion of *Abrus mollis* extract. Compounds showing recovery values exceeding 100% (e.g., *p*-coumaric acid hexoside, isoschaftoside/schaftoside) likely reflect enhanced release of bound or matrix-associated phenolics during in vitro gastrointestinal conditions compared with their extractability from the untreated plant material. In addition, isovitexin, isoorientin, and isoscoparin were confirmed in the EtD (compounds retained in pellet), but only in low proportions (0.56–12.27%). Finally, all identified phenolic compounds found in EtD (compounds retained in pellet) may be potentially available for microbial metabolism in the colon. In this study, the colon phase was not simulated; it was only suggested that compounds contained in the crude digested ethanolic extract (EtD) were derived from the solid pellet of digested leaves, which may potentially become available in the colon phase.

### 3.4. Antioxidant Properties of Control and Digested C. flexuosus Extracts

Considering that phenolic compounds act as antioxidants by reacting with a variety of free radicals, we tested the antioxidant activity of the control and digested *C. flexuosus* extracts as a further step in the evaluation of their bioactivity. The antioxidant activity was evaluated using three complementary assays, and the results are shown in Table 4. Both the ethanolic and aqueous as well as the digested and control extracts exerted dose-dependent antioxidant activity in a wide range of concentrations (from 0.15625 to 10 mg/mL).

In the DPPH assay, the control ethanolic extract showed stronger antioxidant activity than the aqueous extract (IC50 values = 2.23 ± 0.11 vs. 4.30 ± 0.10 mg/mL), especially when compared to the digested extracts, for which IC50 values were not reached. Only the control samples, tested at the highest concentration, had a similar DPPH neutralizing capacity to BHT and ascorbic acid, tested at ten times lower concentrations. The ability of the extracts to scavenge DPPH radicals decreased significantly after the simulated digestion process (by 2.6–4.2, depending on the concentration) (Table 6), so that only three of the highest concentrations of both extracts were active in this assay.

The TRP value of the ethanolic control extract of *C. flexuosus* was higher than that of the aqueous extract at all applied concentrations and reached the value of the tested ascorbic acid at a tenfold lower concentration (10 vs. 1 mg/mL). After digestion, only the two highest concentrations of both the ethanolic and the aqueous extract showed measurable TRP values, which were almost 13–30-fold lower compared to the control extracts (Table 6).

In contrast to the DPPH and TRP assays, the results of the β-carotene bleaching assay showed that the AqC exhibited a stronger antioxidant effect compared to the EtC (IC50 values = 7.46 vs. 13.75 mg/mL). The digested extracts showed lower antioxidant potential than the undigested (control) samples, and IC50 values were not reached for either AgD or AqC. The maximum percentage inhibition by the AqC was 56.15% at a concentration of 10 mg/mL, and this activity was found to be weak compared to ascorbic acid (98.08% at 1 mg/mL). The digested extracts showed 2.7–6.0-fold lower inhibition than the corresponding controls (Table 6) and were active only at the three highest applied concentrations.

The 50% ethanolic fraction obtained from the total extract of the aerial parts of *C. flexuosus* proved to be a better antioxidant compared to the extracts tested in the current study (IC50 values were 146.60 and 195.54 µg/mL for DPPH and ABTS, respectively) [13]. Furthermore, three commercial ethanolic extracts of *Cymbopogon* species (*C. winterianus*, *C. nardus*, and *C. citratus*) showed lower IC50 values in the two mentioned assays compared to our study, which were lower for the leaf extracts (below 100 µg/mL) than for stems and roots [4]. In contrast to our results, Diab et al. [15] observed an increase in antioxidant activity after simulated digestion of the aerial parts of *Th. spicata* by DPPH, ABTS, and FRAP assays, and also found a higher antioxidant activity of the ethanolic extract compared to the aqueous extract, which is in agreement with the results of the DPPH and TRP assays in the present study. Other authors also observed a significant reduction in the antioxidant capacity of fruit extracts rich in phenols [16] and polysaccharides [17] after the digestion process. Given that phenols and flavonoids are considered to be carriers of antioxidant activity, Ma et al. [16] hypothesized that the decrease in antioxidant activity under the influence of gastrointestinal enzymes, pH, and inorganic salts is correlated with that of TPC/TFC. Our results show that some glycosides are detected only in the control samples (compounds **28**, **29**, **31**) as well as caffeic acid (**8**) and its derivatives **13** and **14** (Table 2). Four other hydroxycinnamic acid derivatives (**9**, **17**–**19**) could also contribute to the significantly higher antioxidant capacity of EtD compared to EtC, as they are exclusively present in the ethanolic extracts and remain in low amounts (PRC < 5%) in the pellet (Table 3). Caffeic acid and its derivatives have been shown to be potent antioxidants [48,49]. AgC contained most of the compounds detected in EtC, but most of these compounds were digested during simulated GID. However, the R1/R2 rates of certain compounds were higher than 20% (**12**, **22**, **24**), while two others (**10** and **26**), which were likely released by digestion (Table 3), were previously highlighted for their antioxidant properties [50,51]. With the addition of compounds **7** and **24**, the latter compounds are released during GID but remain in the pellet (PRC > 100%). This leaves open the possibility of digestion of these compounds in the colon after exposure to gut microorganisms, as suggested by Pei et al. [50] for the digestion of coumaric acid conjugates with tartaric acid, glycerol, and glucose.

### 3.5. Antidiabetic Activity of Control and Digested C. flexuosus Extracts

Persistent oxidative stress leads to chronic inflammation, which is responsible for many chronic diseases such as diabetes and cancer. In addition to the antioxidant effect, phenols isolated from plants are able to inhibit α-glucosiase and α-amylase, which play a key role in the digestion of carbohydrates and lead to an increase in blood sugar levels [16]. Both digested and control aqueous and ethanolic extracts showed dose-dependent inhibition of α-glucosidase over a wide range of tested concentrations from 15,625 to 10 mg/mL (Table 5). The extracts after simulated digestion inhibited this enzyme more strongly than the corresponding controls with IC50 values of 4.87 ± 0.22 mg/mL vs. 7.44 ± 0.31 mg/mL for AqD vs. AqC and 4.99 ± 0.11 mg/mL vs. 6.42 ± 0.22 mg/mL for the EtD vs. EtC extracts. The maximum α-glucosidase inhibition was found for AqD at 10 mg/mL (76.93%), which was significantly lower but comparable to the value obtained for the positive control acarbose at a concentration of 1 mg/mL (87.74%). Depending on the concentration, the calculated D/C ratio varied between 0.7 and 1.3, primarily indicating an increase in α-glucosidase inhibitory activity after the simulated digestion procedure (Table 6). In contrast, all tested extracts slightly inhibited α-amylase (2.64–10.09%), which was insignificant compared to the positive control acarbose (88.99%) at a concentration of 1 mg/mL. AgC and EtC were active to some extent at the two highest concentrations tested, while the corresponding digested enzymes only had measurable activity at 10 mg/mL. After digestion, α-amylase inhibitory activity was 3.0- to 3.2-fold lower (Table 6).

In contrast to the low inhibition at high concentrations obtained in the present study (Table 5), Sharma et al. [11] found that the aqueous extract inhibited 16.8% of α-amylase activity at relatively low concentrations of 100 μg/mL. To our knowledge, there are no results available for the α-glucosidase inhibitory activity of *C. flexuosus* extracts. EtD and especially AqD showed increased α-glucosidase inhibitory activity compared to their predigested (control) counterparts (Table 5). Ma et al. [16] also observed an increase in α-glucosidase inhibition in the digested extracts from winter jujube fruit and suggested that the influence of digestive enzymes and pH changes could release additional α-glucosidase inhibitory components. As previously described in Section 3.2, 19 of the total 32 phenolic components were recovered at different R1/R2/PRC rates after simulated intestinal digestion (Table 6). The recovery rate of hydroxybenzoic acid derivatives was up to 26.50%, while among the hydroxycarboxylic acid derivatives, coumaric acid hexanoside (**10**) showed the highest recovery rate (R1/R2/PRC above 100%), followed by coumaroylquinic acid (**11**). Coumaric acid (**7**) was also probably released during digestion and retained in the pellet (PRC = 205.20%). As suggested by Pei et al. [50], coumaric acid and its conjugates delay the intestinal absorption of dietary carbohydrates by binding to glucosidase and subsequently reducing enzymatic activity, and indicated that coumaric acid has a much higher inhibitory effect on this enzyme compared to free coumaric acid. Among the flavonoids, components **22**, **24,** and especially **26** showed the highest recoveries, of which **24** and **26** were released during digestion in the pellet. Tricin glycosides (**22** and **24**) are not yet described in the literature as glucosidase inhibitors, while their aglycone tricin is known to have significant interactions with α-glucosidase and forms conventional hydrogen bonds with multiple glucosidase binding sites [52]. Schaftoside and isoschaftoside (compound **26**) were also shown to be α-glucosidase inhibitors with activity comparable to that of acarbose [53].

**Table 6:** The ratio of TPC, TFC, and biactivities among digested and control extract of *C. flexuosus* at different concentrations.

	Concentration (mg/mL)	Value Ratio D:C	
Extract		Ethanolic	Aqueous
TPC (mg GAE/g)	5	1:8.5	1:9.5
TFC (mg QE/g)	5	1:4.7	1:4.9
DPPH (% of inhibition)	2.5	1:4.0	1:4.2
	5	1:3.6	1:3.9
	10	1:2.6	1:3.8
TRP (µM AAE/g)	5	1:17.3	1:29.9
	10	1:12.9	1:20.7
β-carotene bleaching (% of inhibition)	2.5	1:3.7	1:3.8
	5	1:6.0	1:2.7
	10	1:3.6	1:4.1
α-glucosidase (% of inhibition)	0.15625	1:1.3	1:0.8
	0.3125	1:1.1	1:0.9
	0.625	1:0.8	1:0.8
	1.25	1:0.9	1:0.8
	2.5	1:0.8	1:0.9
	5	1:0.9	1:0.7
	10	1:1	1:0.7
α-amylase (% of inhibition)	10	1:3.2	1:3.0

### 3.6. Cytotoxic Activity of Control and Digested C. flexuosus Extract

To determine the cytotoxicity of the extracts of *C. flexuosus*, healthy human keratinocytes (HaCaT cells) were first exposed to the concentration range (10–250 µg/mL) of the extracts for 24 h and then prolonged for 72 h. None of the extracts (control or digested, ethanol or water) led to a reduction in the viability of healthy human keratinocytes, indicating that these extracts are completely safe for healthy cells and human use, without any cytotoxicity even at the highest tested dose. Consistently, other authors have also reported no significant toxicity toward normal fibroblasts at concentrations below 100 µg/mL [54]. These findings are supported by other authors who also propose lemongrass as a functional food and dietary supplement due to the presence of α-glucosidase inhibitors and its low cytotoxic activity [55].

**Figure 4:**
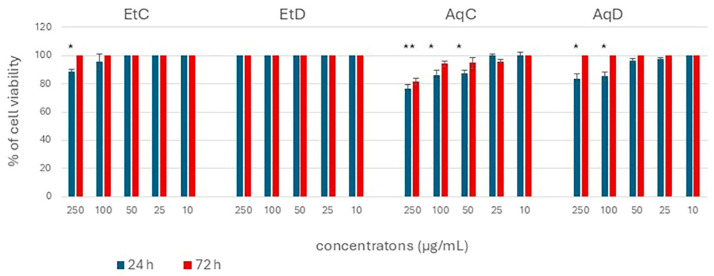
Effect of the tested extracts on the viability of HCT-116 cells. The results are shown as mean ± standard error of three independent experiments. * Statistically significant difference (*p* < 0.05) compared to the control values (100% viable cells).

Subsequently, the colon cancer cell line (HCT-116 cells) was used as a model to determine the anticancer potential of the *C. flexuosus* extracts. Although many studies have demonstrated the antiproliferative and cytotoxic activities of *C. flexuosus* essential oils against a wide range of cancer cell lines [1,9,56], only limited data are available regarding the activity of its extracts [13,54]. Figure 3 shows that a significant reduction in HCT-116 cell viability was observed only at the highest concentrations applied (Figure 4). Non-significant cytotoxicity was generally observed, especially after prolonged exposure (72 h). Also, the IC50 values could not be calculated as none of the applied concentrations killed more than 50% of the cells. The IC50 values above the highest applied concentration (250 µg/mL) indicate a non-cytotoxic and non-carcinogenic activity of these extracts on HCT-116 colon cancer cells. Comparing the observed results between the extracts, water proved to be a better solvent for extraction the secondary metabolites with cytotoxic activity than ethanol, although more total phenol and flavonoid contents were extracted by ethanol (Figure 4). This result emphasizes the importance of the quantitative composition of individual contents in the extract. In addition, the digested extract showed weaker activity compared to the control, as well as a longer exposure time than 24 h. This indicates that the cytotoxic effects observed at high concentrations of the control extracts are due to components present in the control but absent in the digested extracts. Among these components are caffeic acid, which has been well demonstrated to possess anticancer properties [57,58], accompanied by additional compounds with documented cytotoxic activity, such as dicaffeoylquinic acid [59], caffeoylquinic acid [60], isoorientin 4′-O-glucoside [61], and acacetin-6-C-(6″-O-malonyl)glucoside [62] (Table 2). Furthermore, other compounds detected in both the control and digested extracts may be present at comparatively higher concentrations in the control extracts.

Since no IC50 ≤ 250 µg/mL was observed for HCT-116 cells, the maximal effect (Emax) with its 95% confidence interval, calculated for the highest tested concentration of extracts that produced measurable activity, was used to determine the Selectivity Index (SI). The SI was calculated using the formula: SI = Emax (95% CI) for the normal cell line, HaCaT / Emax (95% CI) for the colorectal cancer cell line, HCT-116, only for extracts that showed any activity. The Emax with 95% CI was presented in Table S1, as well as appropriate SIs.

Given the observed low cytotoxicity of the extracts, a low selectivity was consequently observed. Some authors consider selectivity to be good when SI values are greater than 3 [63]. Therefore, although these values are above 1, they do not indicate sufficiently high selectivity.

## 4. Conclusions

This study demonstrated that *Cymbopogon flexuosus* leaf extracts are a valuable source of phenolic acids and flavonoids, with ethanol extracts showing the highest phenolic content and antioxidant capacity. Simulated gastrointestinal digestion significantly reduced TPC, TFC, and antioxidant activity, but the high recovery of compounds such as coumaric acid hexoside, ferulic acid hexoside, and isoschaftoside/schaftoside indicated the release of bound phenolics and their potential colonic availability. Digested extracts, particularly EtD, exhibited enhanced α-glucosidase inhibition, although weaker than acarbose, while all extracts were safe for keratinocytes, and aqueous extracts showed moderate cytotoxicity against colorectal carcinoma cells. Overall, gastrointestinal digestion markedly modulated the bioaccessibility and bioactivity of *C. flexuosus* phenolics, shifting their functional profile from predominantly antioxidant to enhanced antidiabetic potential, while maintaining safety in normal cells and displaying selective anticancer effects, thus supporting their potential application in functional foods and nutraceuticals targeting metabolic health.

## Figures and Tables

**Table 1:** The composition of the buffers used in the simulated digestion. SSF—simulated salivary fluid; SGF—simulated gastric fluid; SIF—simulated intestinal fluid.

Simulated Digestion Fluid	SSF	SGF	SIF
pH	7	3	7
Potassium chloride (0.5 M)	15.1	6.9	6.6
Potassium dihydrogen phosphate (0.5 M)	3.7	0.9	0.8
Sodium hydrogen carbonate (1 M)	6.8	12.5	42.5
Sodium chloride (1.5 M)	-	11.8	9.6
Magnesium chloride hexahydrate (0.15 M)	0.5	0.4	1.1
Sodium carbonate (0.5 M)	0.06	0.5	-

**Table 2:** Identification and characterization of phenolic compounds in control and digested *C. flexuosus* extracts/samples. Tentatively identified compounds, mean expected retention times (RT), molecular formula, calculated mass, *m*/*z* exact mass, mean mass accuracy (mDa), and MS fragments are presented in Table.

No	RT	Tentatively Identified Compounds	Formulas	Calculated Mass	*m*/*z* Exact Mass	mDa	MS Fragments (Main Fragment, %)	Presence of Compounds in Extracts	Ref. **
*Phenolic* *acids*									
*Hydroxybenzoic acid and derivatives*									
**1**	4.24	**Hydroxybenzoic acid**	C_7_H_5_O_3_^−^	137.0239	137.0240	0.13	**108.0212(100)**, 109.0264	EtC, AqC, AqD, EtD	[27]
**2**	2.42	**Dihydroxybenzoic acid is. I** (Protocatehuic acid) *	C_7_H_5_O_4_^−^	153.0188	153.0191	0.32	**108.0212(100)**, 109.0287	EtC, AqC, AqD, EtD	Std.; [38]
**3**	5.80	Dihydroxybenzoic acid is. II (Gentisic acid) *	C_7_H_5_O_4_^−^	153.0188	153.0191	0.32	**109.0288(100)**, 108.0213	EtC, AqC, AqD, EtD	Std.; [38]
**4**	2.51	**Dihydroxybenzoic acid hexoside is. I**	C_13_H_15_O_9_^−^	315.0716	315.0722	0.59	**108.0212(100)**, 109.0285, 152.0106, 153.0176	EtC, AqC, AqD, EtD	-
**5**	3.00	**Dihydroxybenzoic acid hexoside is. II**	C_13_H_15_O_9_^−^	315.0716	315.0722	0.59	**109.0288(100)**, 153.0181, 108.0212, 152.0104	EtC, AqC, AqD, EtD	-
**6**	8.84	**Globulusin B**	C_23_H_29_O_12_^−^	497.1659	497.1662	0.3	**168.0055(100)**, 497.1652, 125.0239, 169.0118, 124.0161, 313.0552, 331.0655, 479.1556	EtC, AqC, AqD, EtD	-
*Hydroxycinnamic acid and derivatives*									
**7**	7.28	Coumaric acid *	C_9_H_7_O_3_^−^	163.0395	163.0397	0.18	**119.0498(100)**, 120.0532, 117.0343	EtC, AqC, AqD, EtD	Std.; [25]
**8**	6.67	Caffeic acid *	C_9_H_7_O_4_^−^	179.0344	179.0346	0.17	**135.0443(100)**, 134.0367, 107.0497	EtC, AqC	Std.; [38]
**9**	9.70	**Ethyl caffeate**	C_11_H_11_O_4_^−^	207.0657	207.0660	0.27	**133.0290(100)**, 135.0444, 134.0358, 161.0236, 179.0346	EtC, EtD	-
**10**	6.37	**Coumaric acid hexoside**	C_15_H_17_O_8_^−^	325.0923	325.0921	−0.24	**119.0497(100)**, 163.0384	EtC, AqC, AqD, EtD	-
**11**	7.58	**Ferulic acid hexoside**	C_16_H_19_O_9_^−^	355.1029	355.1026	−0.31	**193.0497(100)**, 134.0367, 161.0235, 133.0275, 178.0233	AqD, EtD	-
**12**	6.93	**Coumaroylquinic acid**	C_16_H_17_O_8_^−^	337.0923	337.0925	0.16	**191.0549(100)**, 119.0497, 163.0388, 111.0442, 173.0445, 127.0396, 155.0337, 145.0283	EtC, AqC, AqD, EtD	-
**13**	3.72	**Caffeoylquinic acid is. I**	C_16_H_17_O_9_^−^	353.0873	353.0870	−0.26	**191.055(100)**, 135.0444, 179.0338, 173.0444, 161.0230, 155.0343, 127.0393, 111.0444	EtC, AqC	[25,26]
**14**	4.78	**Caffeoylquinic acid is. II**	C_16_H_17_O_9_^−^	353.0873	353.0870	−0.26	**191.0551(100)**, 135.0444, 179.0339, 173.0447, 161.0234, 155.0338, 127.0394, 111.0446	EtC, AqC	[25,26]
**15**	6.27	Caffeoylquinic acid is. III (Chlorogenic acid) *	C_16_H_17_O_9_^−^	353.0873	353.0870	−0.26	**191.0551(100)**, 135.0444, 127.0395, 161.0235, 173.0448	EtC, AqC, AqD, EtD	Std.; [25,26]
**16**	8.37	**Dicaffeoylquinic acid**	C_25_H_23_O_12_^−^	515.119	515.1193	0.35	**179.0340(100)**, 173.0448, 191.055, 353.0864, 135.0445, 111.0088, 161.024, 155.0337, 323.0554	EtC, AqC, AqD	-
**17**	10.46	**1**,**3-*****O*****-Dicoumaroyl-glycerol**	C_21_H_19_O_7_^−^	383.1131	383.1134	0.32	**119.0497(100)**, 163.0393, 145.0290, 197.0811, 219.0645, 237.0751, 297.0402, 311.0552	EtC, EtD	[21]
**18**	9.82	**1**,**3-*****O*****-Coumaroyl-caffeoyl-glycerol**	C_21_H_19_O_8_^−^	399.108	399.1090	1.01	**163.0392(100)**, 119.0496, 135.0444, 145.0289, 179.0339, 253.0707, 235.0600, 217.1065, 297.0731, 327.0949	EtC, EtD	[21]
**19**	10.56	**1**,**3-*****O*****-Coumaroyl-feruloyl-glycerol**	C_22_H_21_O_8_^−^	413.1236	413.1238	0.16	**134.0365(100)**, 163.0391, 119.0497, 145.0290, 193.0498, 175.0395, 219.0662, 234.0522, 267.0849, 337.0340, 398.0992	EtC	[21]
*Flavonoids*									
*Flavone aglycones and derivatives (O-glycosides and flavonolignans)*									
**20**	9.51	Luteolin *	C_15_H_9_O_6_^−^	285.0399	285.0399	−0.01	**133.0290(100)**, 151.0030, 285.0393, 107.0137, 121.0292, 175.0391, 199.0389, 217.0493, 241.0491, 257.0433, 267.0287	EtC, AqC, AqD, EtD	Std.; [27]
**21**	10.25	**Tricin**	C_17_H_13_O_7_^−^	329.0661	329.0663	0.17	**299.0187(100)**, 271.0238, 227.0337, 314.0417, 161.0236, 203.0337, 185.0234, 285.0398	EtC, AqC, AqD, EtD	-
**22**	8.59	**Tricin 7-** ***O*** **-hexoside or Tricin 5-** ***O*** **-hexoside**	C_23_H_25_O_12_^+^	493.1346	493.1353	0.7	**331.0811(100)**, 332.0843, 315.0493, 316.0569, 270.052	EtC, AqC, AqD, EtD	[21]
**23**	10.45	**Tricin 4′-** ***O*** **-(** ***erythro*** **-** ***β*** **-guaiacyl-glyceryl) ether**	C_27_H_25_O_11_^−^	525.1397	525.1402	0.51	**165.0549(100)**, 329.0654, 314.0417, 150.0312, 299.0190, 195.0652, 180.0414, 285.0376, 271.0230, 477.1168	EtC, AqC, EtD	[21]
**24**	8.91	**Tricin 4′-** ***O*** **-(** ***erythro*** **-** ***β*** **-guaiacyl-glyceryl) ether-7-** ***O*** **-hexoside**	C_33_H_35_O_16_^−^	687.1925	687.193	0.49	**329.0649(100)**, 195.0656, 165.0545, 150.0314, 314.0410, 299.0195, 525.1384	EtC, AqC, AqD, EtD	-
*Flavone-C-glycosides (Subgroups of Flavone)*									
**25**	7.88	**Isovitexin** **(Apigenin-6-** ***C*** -glucoside) *	C_21_H_21_O_10_^+^	433.1135	433.1143	0.83	**283.0597(100)**, 313.0704, 271.0598, 295.0622, 323.0912, 337.0708, 349.0705, 361.0706, 379.0812, 397.0918, 415.1026	EtC, AqC, AqD, EtD	Std.
**26**	7.32	**Isoschaftoside or Schaftoside** **(Apigenin-6**,**8-*****C*****-pentoside hexoside)**	C_26_H_29_O_14_^+^	565.1557	565.1563	0.57	**379.0813(100)**, 325.0700, 409.0911, 295.0598, 337.0700, 355.0809, 445.0999, 475.1034, 457.1037, 451.1031, 493.1121, 511.1230	EtC, AqC, AqD, EtD	[21,25]
**27**	7.47	**Isoorientin** **(Luteolin-6-** ***C*** **-glucoside)**	C_21_H_21_O_11_^+^	449.1084	449.1099	1.51	**299.0548(100)**, 329.0654, 287.0548, 325.0698, 339.0856, 353.0652, 365.0661, 377.0659, 395.0766, 413.0868, 431.0975	EtC, AqC, AqD, EtD	[25,27]
**28**	7.19	**Isocarlinoside or Carlinoside** **(Luteolin-6**,**8-*****C*****-pentoside hexoside)**	C_26_H_29_O_15_^+^	581.1506	581.1514	0.75	**395.0761(100)**, 425.0868, 341.0652, 443.0961, 407.0761, 413.0864, 353.0656, 491.0989, 461.0949, 527.1186, 509.1075, 449.0871, 467.0975	EtC, AqC	[21,25]
**29**	7.00	**Isoorientin 4′-** ***O*** **-glucoside**	C_27_H_31_O_16_^+^	611.1612	611.1624	1.19	**329.0652(100)**, 299.0547, 353.0654, 311.0541, 287.0547, 383.0757, 395.0761, 413.0868, 431.0970, 449.1063	EtC, AqC	-
**30**	8.05	**Isoscoparin** **(Chrysoeriol-6-** ***C*** **-glucoside)**	C_22_H_23_O_11_^+^	463.124	463.1249	0.86	**313.0710(100)**, 343.0815, 367.0813, 325.0721, 339.0848, 353.1013, 367.0813, 379.0810, 391.0810, 409.0921, 427.1025, 445.1121	EtC, AqC, EtD	[27]
**31**	8.05	**Acacetin-6-** ***C*** **-(6”-** ***O*** **-malonyl)glucoside**	C_25_H_23_O_13_^−^	531.1139	531.1146	0.73	**339.0497(100)**, 191.0551, 229.0129, 295.0569, 327.0508, 159.0448, 199.0968, 357.0602, 487.127	EtC, AqC	-
*Other compounds*									
**32**	0.61	**Quinic acid**	C_7_H_11_O_6_^−^	191.0556	191.0555	−0.06	**109.0287(100)**, 127.0388, 191.0545, 171.0279, 137.0224, 173.0457	EtC, AqC, AqD, EtD	[21]

Abbreviations: Std—Standard; Is.—Isomer; * Compounds confirmed by comparison with available standards. ** Compounds previously reported and found in various Cymbopogan species (*Cymbopogan citratus*) with representative references.

**Table 3:** Recovery (%) of all identified *C. flexuosus* phenolic compounds, after in vitro GID, using UHPLC Q-ToF MS.

No.	Tentatively Identified Compounds	Bioaccessibility (Recovery)		
		R1, %	R2, %	PRC, %
Hydroxybenzoic acid and derivatives				
1	Hydroxybenzoic acid	16.07	6.52	8.89
2	Dihydroxybenzoic acid is. I (Protocatehuic acid)	2.02	2.20	2.69
3	Dihydroxybenzoic acid is. II (Gentisic acid)	4.24	4.07	15.05
4	Dihydroxybenzoic acid hexoside is. I	12.03	14.76	24.42
5	Dihydroxybenzoic acid hexoside is. II	7.87	10.67	26.50
6	Globulusin B	2.13	1.95	4.59
Hydroxycinnamic acid and derivatives				
7	Coumaric acid	10.12	9.02	205.20
8	Caffeic acid	0	0	0
9	Ethyl caffeate	-	0	3.24
10	Coumaric acid hexoside	100.81	113.97	134.32
11	Ferulic acid hexoside	-	-	-
12	Coumaroylquinic acid	46.05	63.24	43.81
13	Caffeoylquinic acid is. I	0	0	0
14	Caffeoylquinic acid is. II	0	0	0
15	Caffeoylquinic acid is. III (Chlorogenic acid)	0.73	0.75	0.57
16	Dicaffeoylquinic acid	19.43	8.12	0
17	1,3-*O*-Dicoumaroyl-glycerol	-	0	4.96
18	1,3-*O*-Coumaroyl-caffeoyl-glycerol	-	0	4.95
19	1,3-*O*-Coumaroyl-feruloyl-glycerol	-	0	0
Flavone aglycones and derivatives				
20	Luteolin	4.29	0.65	2.78
21	Tricin	4.07	0.61	13.76
22	Tricin 7-*O*-hexoside or Tricin 5-*O*-hexoside	68.81	24.84	77.38
23	Tricin 4′-*O*-(*erythro*-*β*-guaiacyl-glyceryl)ether	0	0	12.24
24	Tricin 4′-*O*-(*erythro*-*β*-guaiacyl-glyceryl)ether-7-*O*-hexoside	86.91	39.44	120.56
Flavone-C-glycosides (Subgroups of Flavone)				
25	Isovitexin (Apigenin-6-*C*-glucoside)	2.97	1.34	12.27
26	Isoschaftoside or Schaftoside (Apigenin-6,8-*C*-pentoside hexoside)	110.11	100.84	114.64
27	Isoorientin (Luteolin-6-*C*-glucoside)	0.48	0.28	0.56
28	Isocarlinoside or Carlinoside (Luteolin-6,8-*C*-pentoside hexoside)	0	0	0
29	Isoorientin 4′-*O*-glucoside	0	0	0
30	Isoscoparin (Chrysoeriol-6-*C*-glucoside)	0	0	5.12
31	Acacetin-6-*C*-(6″-*O*-malonyl)glucoside	0	0	0
Other compounds				
32	Quinic acid	20.98	16.66	18.91

Abbreviations: R1, R2, and PRC were explained in Section 2.5. (see this subsection). “-“ compound not detect in control ethanolic and/or aqueous extracts, only was found in digested extracts. If recovery and/or pellet retained are “0”, compound was not detect in digested aqueous extract and/or digested ethanolic extract and was only found in control extracts; when recovery and/or the proportions of compound retained in pellet exceed 100%, this suggests that the compound was likely liberated from *C. flexuosus* leaves during in vitro GID. These compounds appear to show greater release from the digested plant material under gastrointestinal conditions compared with extraction from the initial plant material.

**Table 4:** Antioxidant activity of digested and control aqueous and ethanolic extracts of *C. flexuosus* at different concentrations.

Samples	Concentration [mg/mL]	DPPH Assay [% of Inhibition]	TRP Assay [µM AAE/g]	β-Carotene Bleaching Assay [% of Inhibition]
Extracts				
AqC	0.15625	12.07 ± 0.43 ^d^	3.67 ± 0.50 ^c^	na
	0.3125	16.69 ± 0.43 ^d^	6.90 ± 0.50 ^c^	na
	0.625	23.29 ± 1.96 ^d^	12.76 ± 1.95 ^d^	11.71 ± 1.50 ^b^
	1.25	32.09 ± 1.53 ^d^	22.71 ± 1.86 ^d^	17.06 ± 1.82 ^b^
	2.5	42.75 ± 2.12 ^d^	41.67 ± 1.53 ^c^	21.03 ± 1.82 ^b^
	5	59.45 ± 1.54 ^d^	66.90 ± 2.42 ^d^	26.59 ± 1.82 ^b^
	10	87.03 ± 0.59 ^ab^	111.81 ± 3.77 ^c^	56.15 ± 2.48 ^b^
IC50 (mg/mL)		4.30 ± 0.10	-	7.46 ± 0.32
AqD	0.15625	na	na	na
	0.3125	na	na	na
	0.625	na	na	na
	1.25	na	na	na
	2.5	10.27 ± 1.38 ^e^	na	5.56 ± 0.91 ^c^
	5	15.23 ± 1.34 ^e^	2.24 ± 0.81 ^e^	9.72 ± 0.91 ^c^
	10	23.01 ± 1.92 ^d^	5.71 ± 0.29 ^d^	13.69 ± 1.57 ^d^
IC50 (mg/mL)		nd	-	nd
EtC	0.15625	29.61 ± 0.94 ^c^	5.19 ± 0.22 ^c^	na
	0.3125	36.66 ± 1.57 ^c^	9.33 ± 0.44 ^c^	na
	0.625	44.05 ± 1.27 ^c^	17.48 ± 0.93 ^c^	5.75 ± 0.69 ^b^
	1.25	48.45 ± 1.36 ^c^	27.62 ± 0.68 ^c^	8.53 ± 1.50 ^c^
	2.5	55.61 ± 2.14 ^c^	42.33 ± 1.79 ^c^	14.68 ± 1.50 ^b^
	5	69.04 ± 0.90 ^c^	75.00 ± 2.02 ^c^	21.03 ± 2.08 ^b^
	10	88.16 ± 2.15 ^ab^	127.10 ± 2.27 ^b^	37.50 ± 2.15 ^c^
IC50 (mg/mL)		2.23 ± 0.11	-	13.75 ± 0.88
EtD	0.15625	na	na	na
	0.3125	na	na	na
	0.625	na	na	na
	1.25	na	na	na
	2.5	13.99 ± 0.93 ^e^	na	3.97 ± 0.91 ^c^
	5	19.01 ± 0.93 ^f^	4.33 ± 0.30 ^e^	5.16 ± 0.91 ^c^
	10	33.39 ± 1.44 ^c^	9.86 ± 0.62 ^d^	10.32 ± 1.24 ^d^
IC50 (mg/mL)		nd	-	nd
Positive controls				
BHT	1	84.10 ± 0.15 ^b^	138.86 ± 1.51 ^a^	>100
Ascorbic acid	1	88.26 ± 0.10 ^a^	129.71 ± 1.51 ^b^	98.08 ± 6.17 ^a^

For each tested concentration, mean values with different superscript letters (a–f) are significantly different (one-way ANOVA, Tukey’s post hoc; *p* < 0.05). The superscript letters indicate the values in descending order, with (a) representing the highest value; na—no activity, nd—not determined; (-)—not applicable.

**Table 5:** Antidiabetic activity of digested and control aqueous and ethanolic extracts of *C. flexuosus*, at different concentrations.

Samples	Concentration [mg/mL]	α-Glucosidase [% of Inhibition]	α-Amylase [% of Inhibition]
Extracts			
AqC	0.15625	10.25 ± 0.42 ^c^	na
	0.3125	13.99 ± 1.20 ^c^	na
	0.625	20.90 ± 0.67 ^c^	na
	1.25	24.87 ± 0.91 ^d^	na
	2.5	33.76 ± 0.97 ^c^	na
	5	41.05 ± 1.03 ^e^	4.52 ± 0.97 ^b^
	10	58.89 ± 2.20 ^c^	8.23 ± 0.84 ^c^
IC50 (mg/mL)		7.44 ± 0.31	nd
AqD	0.15625	13.14 ± 0.66 ^c^	na
	0.3125	15.51 ± 0.69 ^c^	na
	0.625	25.52 ± 0.51 ^b^	na
	1.25	31.41 ± 0.98 ^bc^	na
	2.5	39.53 ± 1.32 ^b^	na
	5	55.15 ± 0.99 ^b^	na
	10	79.15 ± 3.39 ^b^	2.64 ± 0.18 ^d^
IC50 (mg/mL)		4.87 ± 0.22	nd
EtC	0.15625	15.93 ± 0.34 ^b^	na
	0.3125	20.31 ± 1.16 ^b^	na
	0.625	21.21 ± 1.06 ^c^	na
	1.25	28.95 ± 1.04 ^c^	na
	2.5	35.47 ± 0.97 ^c^	na
	5	47.50 ± 1.04 ^d^	5.80 ± 0.68 ^b^
	10	63.56 ± 2.06 ^c^	10.09 ± 0.74 ^b^
IC50 (mg/mL)		6.42 ± 0.22	nd
EtD	0.15625	11.86 ± 0.95 ^cd^	na
	0.3125	18.42 ± 0.36 ^b^	na
	0.625	26.27 ± 1.20 ^b^	na
	1.25	33.37 ± 1.18 ^b^	na
	2.5	42.04 ± 1.52 ^b^	na
	5	51.31 ± 0.62 ^c^	na
	10	76.93 ± 1.79 ^b^	3.20 ± 0.37 ^d^
IC50 (mg/mL)		4.99 ± 0.11	nd
Positive control			
Acarbose	1	87.74 ± 0.25 ^a^	82.99 ± 1.18 ^a^

For each tested concentration, mean values with different superscript letters (a–e) are significantly different (one-way ANOVA, Tukey’s post hoc; *p* < 0.05). The superscript letters indicate the values in descending order, with (a) representing the highest value; na—no activity, nd—not determined.

## Data Availability

The original contributions presented in the study are included in the article/Supplementary Material; further inquiries can be directed to the corresponding authors.

## Supplementary Materials

The following supporting information can be downloaded at: https://www.mdpi.com/article/10.3390/foods14223868/s1, Figure S1. Fragmentation patterns (MS/MS spectra) of identified phenolic acids and derivatives: (**1**) Hydroxybenzoic acid; (**2**) Dihydroxybenzoic acid is. I (Protocatehuic acid); (**3**) Dihydroxybenzoic acid is. II (Gentisic acid); (**4**) Dihydroxybenzoic acid hexoside is. I; (**5**) Dihydroxybenzoic acid hexoside is. II; (**6**) Globulsin B; (**7**) Coumaric acid; (**8**) Caffeic acid; (**9**) Ethyl caffeate; (**10**) Coumaric acid hexoside; (**11**) Ferulic acid hexoside; (**12**) Coumaroylquinic acid; (**13**) Caffeoylquinic acid is. I; (**14**) Caffeoylquinic acid is. II; (**15**) Caffeoylquinic acid is. III (Chlorogenic acid); (**16**) Dicaffeoylquinic acid; (**17**) 1,3-*O*-Dicoumaroyl-glycerol; (**18**) 1,3-*O*-Coumaroyl-caffeoyl-glycerol; (**19**) 1,3-*O*-Coumaroyl-feruloyl-glycerol.; Figure S2. Fragmentation patterns (MS/MS spectra) of identified flavonoids and derivatives, and quinic acid: (**20**) Luteolin; (**21**) Tricin; (**22**) Tricin 7-*O*-hexoside or Tricin 5-*O*-hexoside*; (**23**) Tricin 4′-*O*-(*erythro*-β-guaiacyl-glyceryl)ether; (**24**) Tricin 4′-*O*-(*erythro*-β-guaiacyl-glyceryl) ether-7-*O*-hexoside; (**25**) Isovitexin (Apigenin-6-*C*-glucoside)*; (**26**) Isoschaftoside or Schaftoside (Apigenin-6,8-*C*-pentoside hexoside)*; (**27**) Isoorientin (Luteolin-6-*C*-glucoside)*; (**28**) Isocarlinoside or Carlinoside (Luteolin-6,8-*C*-pentoside hexoside)*; (**29**) Isoorientin 4′-*O*-glucoside*; (**30**) Isoscoparin (Chrysoeriol-6-*C*-glucoside)*; (**31**) Acacetin-6-*C*-(6”-*O*-malonyl)glucoside; (**32**) Quinic acid. *Compounds (MS/MS spectra) recorded in positive ionization mode.; Figure S3. The DPPH (**a**,**b**) and β-caroten bleaching activity (**c**,**d**) of the control extracts at different concentrations (**a**,**c**—AqC, **b**,**d**—EtC). Note: The *x*-axis shows the concentration of sample (mg/mL), and the *y*-axis shows the percentage value of inhibition (%). Figure S4. MS-Base peak chromatograms of: (**a**,**e**) Undigested (control) ethanolic extract (EtC); (**b**,**f**) Undigested (control) aqueous extract (AqC); (**c**,**g**) Crude digested aqueous extract (AqD); (**d**,**h**) Crude digested ethanolic extract (EtD); in positive ionization mode (**a**–**d**) and negative ionization mode (**e**–**h**); for peak annotation see retention time in Table 2; Table S1. Emax with 95% CI for the highest tested concentration of extracts that produced measurable activity on colorectal cancer cells, and an appropriate CI for these extracts.

## References

[1] Sharma P.R., Mondhe D.M., Muthiah S., Pal H.C., Shahi A.K., Saxena A.K., Qazi G.N. (2009). Anticancer activity of an essential oil from *Cymbopogon flexuosus*. Chem. Biol. Interact..

[2] Ganjewala D.E., Gupta A.K. (2013). Lemongrass (*Cymbopogon flexuosus* Steud.) Wats essential oil: Overview and biological activities. Recent. Prog. Med. Plants.

[3] Tibenda J.J., Yi Q., Wang X., Zhao Q. (2022). Review of phytomedicine, phytochemistry, ethnopharmacology, toxicology, and pharmacological activities of *Cymbopogon* genus. Front. Pharmacol..

[4] Wahyuni D.K., Kharisma V.D., Murtadlo A.A.A., Rahmawati C.T., Syukriya A.J., Prasongsuk S., Subramaniam S., Wibowo A.T., Purnobasuki H. (2024). The antioxidant and antimicrobial activity of ethanolic extract in roots, stems, and leaves of three commercial *Cymbopogon* species. BMC Complement. Med. Ther..

[5] Skaria B.P., Joy P.P., Mathew G., Mathew S., Joseph A., Peter K.V. (2012). Lemongrass. Handbook of Herbs and Spices.

[6] Lonkar P.B., Chavan U.D., Pawar V.D., Bansode V.V., Amarowicz R. (2013). Studies on preparation and preservation of lemongrass (*Cymbopogon flexuosus* (Steud) Wats) powder for tea. Emir. J. Food Agric..

[7] Nwauche K.T., Berezi E.P., Okari K.A. (2024). Phytochemical profiling of *Cymbopogon flexuosus* plant leaves. Int. J. Sci. Res. Arch..

[8] Adukwu E.C., Bowles M., Edwards-Jones V., Bone H. (2016). Antimicrobial activity, cytotoxicity and chemical analysis of lemongrass essential oil (*Cymbopogon flexuosus*) and pure citral. Appl. Microbiol. Biotechnol..

[9] Caballero-Gallardo K., Quintero-Rincón P., Stashenko E.E., Olivero-Verbel J. (2022). Photoprotective agents obtained from aromatic plants grown in Colombia: Total phenolic content, antioxidant activity, and assessment of cytotoxic potential in cancer cell lines of *Cymbopogon flexuosus* L. and *Tagetes lucida* Cav. essential oils. Plants.

[10] Júnior A.S.S., Aidar F.J., Silva L.A.S., Silva T.d.B., de Almeida S.F.M., Teles D.C.S., Junior W.d.L., Schimieguel D.M., de Souza D.A., Nascimento A.C.S. (2024). Influence of lemongrass essential oil (*Cymbopogon flexuosus*) supplementation on diabetes in rat model. Life.

[11] Sharma R., Pokhrel G., Bhattarai M., Roy K. (2021). Anti-diabetic activity of aqueous extract of *Cymbopogon flexuosus* in high fat diet induced obese guinea pigs. Int. J. Pharm. Res. Appl..

[12] Nomier Y., Asaad G.F., Alshahrani S., Safhi S., Medrba L., Alharthi N., Rehman Z., Alhazmi H., Sanobar S. (2021). Antidepressant and anxiolytic profiles of *Cymbopogon flexuosus* ethanolic extract in chronic unpredictable mild stress induced in rats. Biomed. Pharmacol. J..

[13] Le Q.U., Lay H.L., Wu M.C. (2019). The isolation, structural characterization, and anticancer activity from the aerial parts of *Cymbopogon flexuosus*. J. Food Biochem..

[14] Bellesia A., Verzelloni E., Tagliazucchi D. (2015). Pomegranate ellagitannins inhibit α-glucosidase activity in vitro and reduce starch digestibility under simulated gastro-intestinal conditions. Int. J. Food Sci. Nutr..

[15] Diab F., Khalil M., Lupidi G., Zbeeb H., Salis A., Damonte G., Bramucci M., Portincasa P., Vergani L. (2022). Influence of simulated in vitro gastrointestinal digestion on the phenolic profile, antioxidant, and biological activity of *Thymbra spicata* L. extracts. Antioxidants.

[16] Ma Y.L., Wang Z.T., Yang D.M., Li Z., Wu Q.L., Guo X., Shang Y.F., Thakur K., Wei Z.J. (2024). Effect of processing and simulated digestion on phenolics, antioxidant and hypoglycemic potential of winter jujube. LWT.

[17] Kasipandi M., Manikandan A., Sreeja P.S., Suman T., Saikumar S., Dhivya S., Parimelazhagan T. (2019). Effects of in vitro simulated gastrointestinal digestion on the antioxidant, α-glucosidase and α-amylase inhibitory activities of water-soluble polysaccharides from *Opilia amentacea* Roxb fruit. LWT.

[18] Singleton V.L., Rossi J.A. (1965). Colorimetry of total phenolics with phosphomolybdic-phosphotungstic acid reagents. Am. J. Enol. Vitic..

[19] Park Y.K., Koo M.H., Ikegaki M., Contado J.O.S.E. (1997). Comparison of the flavonoid aglycone contents of *Apis mellifera* propolis from various regions of Brazil. Arq. Biol. Tecnol..

[20] Milinčić D.D., Vidović B.B., Gašić U.M., Milenković M., Kostić A.Ž., Stanojević S.P., Ilić T., Pešić M.B. (2024). A systematic UHPLC Q-ToF MS approach for the characterization of bioactive compounds from freeze-dried red goji berries (*L. barbarum* L.) grown in Serbia: Phenolic compounds and phenylamides. Food Chem..

[21] Aly O., Mekky R., Pereira F., Diab Y., Tammam M., El-Demerdash A. (2024). Deciphering the potential of *Cymbopogon citratus* (DC.) Stapf as an anti-obesity agent: Phytochemical profiling, in vivo evaluations and molecular docking studies. Food Funct..

[22] Lan W., Rencoret J., Lu F., Karlen S.D., Smith B.G., Harris P.J., Del Río J.C., Ralph J. (2016). Tricin-lignins: Occurrence and quantitation of tricin in relation to phylogeny. Plant J..

[23] Boulekbache-Makhlouf L., Meudec E., Mazauric J.P., Madani K., Cheynier V. (2013). Qualitative and semi-quantitative analysis of phenolics in Eucalyptus globulus leaves by high-performance liquid chromatography coupled with diode array detection and electrospray ionisation mass spectrometry. Phytochem. Anal..

[24] Bylka W., Frański R., Stobiecki M. (2002). Differentiation between isomeric acacetin-6-C-(6″-O-malonyl)glucoside and acacetin-8-C-(6″-O-malonyl)glucoside by using low-energy CID mass spectra. J. Mass. Spectrom..

[25] Costa G., Ferreira J.P., Vitorino C., Pina M.E., Sousa J.J., Figueiredo I.V., Batista M.T. (2016). Polyphenols from *Cymbopogon citratus* leaves as topical anti-inflammatory agents. J. Ethnopharmacol..

[26] Costa G., Nunes F., Vitorino C., Sousa J., Figueiredo I., Batista M. (2015). Validation of a RP-HPLC method for quantitation of phenolic compounds in three different extracts from *Cymbopogon citratus*. Res. J. Med. Plant.

[27] Du X., Zhang M., Wang S., Li J., Zhang J., Liu D. (2024). Ethnopharmacology, chemical composition and functions of *Cymbopogon citratus*. Chin. Herb. Med..

[28] Shao S.Y., Ting Y., Wang J., Sun J., Guo X.F. (2020). Characterization and identification of the major flavonoids in *Phyllostachys edulis* leaf extract by UPLC–QTOF–MS/MS. Acta Chromatogr..

[29] Singh A., Kumar S., Bajpai V., Reddy T.J., Rameshkumar K.B., Kumar B. (2015). Structural characterization of flavonoid C-and O-glycosides in an extract of *Adhatoda vasica* leaves by liquid chromatography with quadrupole time-of-flight mass spectrometry. Rapid Commun. Mass. Spectrom..

[30] Blois M.S. (1958). Antioxidant determinations by the use of a stable free radical. Nature.

[31] Tusevski O., Kostovska A., Iloska A., Trajkovska L., Simic S.G. (2014). Phenolic production and antioxidant properties of some Macedonian medicinal plants. Cent. Eur. J. Biol..

[32] Dapkevicius A., Venskutonis R., van Beek T.A., Linssen J.P. (1998). Antioxidant activity of extracts obtained by different isolation procedures from some aromatic herbs grown in Lithuania. J. Sci. Food Agric..

[33] Zengin G., Uysal A., Gunes E., Aktumsek A. (2014). Survey of phytochemical composition and biological effects of three extracts from a wild plant (*Cotoneaster nummularia* Fisch. et Mey.): A potential source for functional food ingredients and drug formulations. PLoS ONE.

[34] Wan L.S., Min Q.X., Wang Y.L., Yue Y.D., Chen J.C. (2013). Xanthone glycoside constituents of *Swertia kouitchensis* with α-glucosidase inhibitory activity. J. Nat. Prod..

[35] Milutinović M., Čurović D., Nikodijević D., Vukajlović F., Predojević D., Marković S., Pešić S. (2020). The silk of *Plodia interpunctella* as a potential biomaterial and its cytotoxic effect on cancer cells. Anim. Biotechnol..

[36] Mosmann T. (1983). Rapid colorimetric assay for cellular growth and survival: Application to proliferation and cytotoxicity assays. J. Immunol. Methods.

[37] Hammer O., Harper D.T., Ryan P.D. (2001). PAST: Paleontological statistics software package for education and data analysis. Palaeontol. Electron..

[38] Muala W.C.B., Desobgo Z.S.C., Jong N.E. (2021). Optimization of extraction conditions of phenolic compounds from *Cymbopogon citratus* and evaluation of phenolics and aroma profiles of extract. Heliyon.

[39] Colombo R., Yariwake J., McCullagh M. (2008). Study of C- and O-glycosylflavones in sugarcane extracts using liquid chromatography—Exact mass measurement mass spectrometry. Artic. J. Braz. Chem. Soc..

[40] El-Seedi H.R., Taher E.A., Sheikh B.Y., Anjum S., Saeed A., AlAjmi M.F., Moustafa M.S., Al-Mousawi S.M., Farag M.A., Hegazy M.-E.F., Attaur R. (2018). Chapter 8—Hydroxycinnamic Acids: Natural Sources, Biosynthesis, Possible Biological Activities, and Roles in Islamic Medicine. Studies in Natural Products Chemistry.

[41] Cheynier V., Comte G., Davies K.M., Lattanzio V., Martens S. (2013). Plant phenolics: Recent advances on their biosynthesis, genetics, and ecophysiology. Plant Physiol. Biochem..

[42] Eudes A., Dutta T., Deng K., Jacquet N., Sinha A., Benites V.T., Baidoo E.E.K., Richel A., Sattler S.E., Northen T.R. (2017). SbCOMT (Bmr12) is involved in the biosynthesis of tricin-lignin in sorghum. PLoS ONE.

[43] Pešić M.B., Milinčić D.D., Kostić A.Ž., Stanisavljević N.S., Vukotić G.N., Kojić M.O., Gašić U.M., Barać M.B., Stanojević S.P., Popović D.A. (2019). In vitro digestion of meat-and cereal-based food matrix enriched with grape extracts: How are polyphenol composition, bioaccessibility and antioxidant activity affected?. Food Chem..

[44] Wojtunik-Kulesza K., Oniszczuk A., Oniszczuk T., Combrzyński M., Nowakowska D., Matwijczuk A. (2020). Influence of in vitro digestion on composition, bioaccessibility and antioxidant activity of food polyphenols—A non-systematic review. Nutrients.

[45] Lai X., Li X., Chen J., Liu X., Pan P., Zhou Y., Zhao G. (2025). Advances in flavonoid glycosylation: Chemical and biological basis, mechanisms, physicochemical properties, and applications in the food industry. Trends Food Sci. Technol..

[46] Cattivelli A., Zannini M., De Angeli M., D’Arca D., Minischetti V., Conte A., Tagliazucchi D. (2024). Bioaccessibility of flavones, flavanones, and flavonols from vegetable foods and beverages. Biology.

[47] Shen W., Hu X., Niu Y., Lu Y., Wang B., Wang H. (2021). Bioaccessibility and absorption of flavonoid C-glycosides from *Abrus mollis* using simulated digestion, Caco-2 cell, and in situ single-pass perfusion models. Planta Med..

[48] de Armas-Ricard M., Ruiz-Reyes E., Ramírez-Rodríguez O. (2019). Caffeates and caffeamides: Synthetic methodologies and their antioxidant properties. Int. J. Med. Chem..

[49] Islam S., Adam Z., Akanda J.H. (2024). Quinic and caffeic acids derivatives: Affecting antioxidant capacities and phenolics contents of certain therapeutic and specialty crops employing water and ethanolic extracts. Food Chem. Adv..

[50] Pei K., Ou J., Huang J., Ou S. (2016). p-Coumaric acid and its conjugates: Dietary sources, pharmacokinetic properties and biological activities. J. Sci. Food Agric..

[51] Lee Y.H., So B.H., Lee K.S., Kuk M.U., Park J.H., Yoon J.H., Lee Y.J., Kim D.Y., Kim M.S., Kwon H.W. (2024). Identification of cellular isoschaftoside-mediated anti-senescence mechanism in RAC2 and LINC00294. Molecules.

[52] Zaripova M., Abdullaev I., Bogbekov A., Gayibov U., Omonturdiev S., Makhmudov R., Ergashev N., Jabbarova G., Gayibova S., Aripov T. (2025). In vitro and in silico studies of *Gnaphalium* U. extract: Inhibition of α-amylase and α-glucosidase as a potential strategy for metabolic syndrome regulation. Trends Sci..

[53] de Oliveira A.P., Coppede J.S., Bertoni B.W., Crotti A.E., França S.C., Pereira A.M.S., Taleb-Contini S.H. (2018). *Costus spiralis* (Jacq.) Roscoe: A novel source of flavones with α-glycosidase inhibitory activity. Chem. Biodivers..

[54] Dwivedi M. (2024). Phytochemical characterization and biological evaluation of lemongrass (*Cymbopogon citratus*) extracts: A systematic experimental study. Int. J. Pharm. Chem. Anal..

[55] Santoso F., Winarno J., Gunawan-Puteri M. (2018). Application of lemongrass (*Cymbopogon citratus*) as a functional food ingredient with alpha-glucosidase inhibitory activity. Proceedings of the 4th International Conference on Food, Agriculture and Natural Resources (FANRes 2018).

[56] Gaonkar R., Shiralgi Y., Lakkappa D.B., Hegde G. (2018). Essential oil from *Cymbopogon flexuosus* as the potential inhibitor for HSP90. Toxicol. Rep..

[57] Alam M., Ahmed S., Elasbali A.M., Adnan M., Alam S., Hassan M.I., Pasupuleti V.R. (2022). Therapeutic implications of caffeic acid in cancer and neurological diseases. Front. Oncol..

[58] Cortez N., Villegas C., Burgos V., Cabrera-Pardo J.R., Ortiz L., González-Chavarría I., Nchiozem-Ngnitedem V.-A., Paz C. (2024). Adjuvant properties of caffeic acid in cancer treatment. Int. J. Mol. Sci..

[59] Lodise O., Patil K., Karshenboym I., Prombo S., Chukwueke C., Pai S.B. (2019). Inhibition of prostate cancer cells by 4,5-Dicaffeoylquinic acid through cell cycle arrest. Prostate Cancer.

[60] Indy Tamayose C., Dos Santos E.A., Roque N., Costa-Lotufo L.V., Pena Ferreira M.J. (2019). Caffeoylquinic acids: Separation method, antiradical properties and cytotoxicity. Chem. Biodivers..

[61] Laurindo L.F., Pomini K.T., de Lima E.P., Laurindo L.F., Rodrigues V.D., da Silva Camarinha Oliveira J., Araújo A.C., Guiguer E.L., Rici R.E.G., Maria D.A. (2025). Isoorientin: Unveiling the hidden flavonoid’s promise in combating cancer development and progression—A comprehensive review. Life Sci..

[62] Li J., Zhong X., Zhao Y., Shen J., Xiao Z., Pilapong C. (2024). Acacetin inhibited non-small-cell lung cancer (NSCLC) cell growth via upregulating miR-34a in vitro and in vivo. Sci. Rep..

[63] Weerapreeyakul N., Nonpunya A., Barusrux S., Thitimetharoch T., Sripanidkulchai B. (2012). Evaluation of the Anticancer Potential of Six Herbs against a Hepatoma Cell Line. Chin. Med..

