# Supplementary material for: Effect of Simulated Gastrointestinal Digestion on the Phenolic Composition and Bioactivity of Cymbopogon flexuosus Extracts
Source: Foods. 2025 Nov 12;14(22):3868. doi: 10.3390/foods14223868 (PMC12651865; doi:10.3390/foods14223868)
Supplement: Supplementary file 1 [file foods-14-03868-s001.zip › foods-3923223-supplementary.pdf]

foods

MDPI

Effect of simulated gastrointestinal digestion on the phenolic composition and bioactivity of *Cymbopogon flexuosus* extracts

Ana Alimpić Aradski[1,*], Danijel D. Milinčić[2], Mirjana B. Pešić[2], Milena Milutinović[3], Eisuke Kuraya[4], Akiko Touyama[4], Danka Bukvički[1,*]

[1] University of Belgrade, Faculty of Biology, Institute of Botany and Botanical Garden „Jevremovac“, Chair of Morphology and Systematics of Plants, Studentski trg 16, Belgrade, Serbia; alimpic.ana@bio.bg.ac.rs (A.A.A.); dankabukvicki@bio.bg.ac.rs (D.B.)
[2] University of Belgrade, Faculty of Agriculture, Institute of Food Technology and Biochemistry, Department of Chemistry and Biochemistry, Nemanjina 6, 11080 Belgrade, Serbia; milincic93@gmail.com (D.D.M.); mpesic@agrif.bg.ac.rs (M.B.P.)
[3] University of Kragujevac, Faculty of Science, Department for Biology and Ecology, Radoja Domanovića 12, 34000 Kragujevac, Serbia; milena.milutinovic@pmf.kg.ac.rs (M.M.)
[4] National Institute of Technology, Okinawa College, 905 Henoko, Nago City, Okinawa 905-2192, Japan, kuraya@okinawa-ct.ac.jp, touyama@flora.okinawa
*Correspondence: dankabukvicki@bio.bg.ac.rs (D.B.); alimpic.ana@bio.bg.ac.rs (A.A.A.);

foods

MDPI

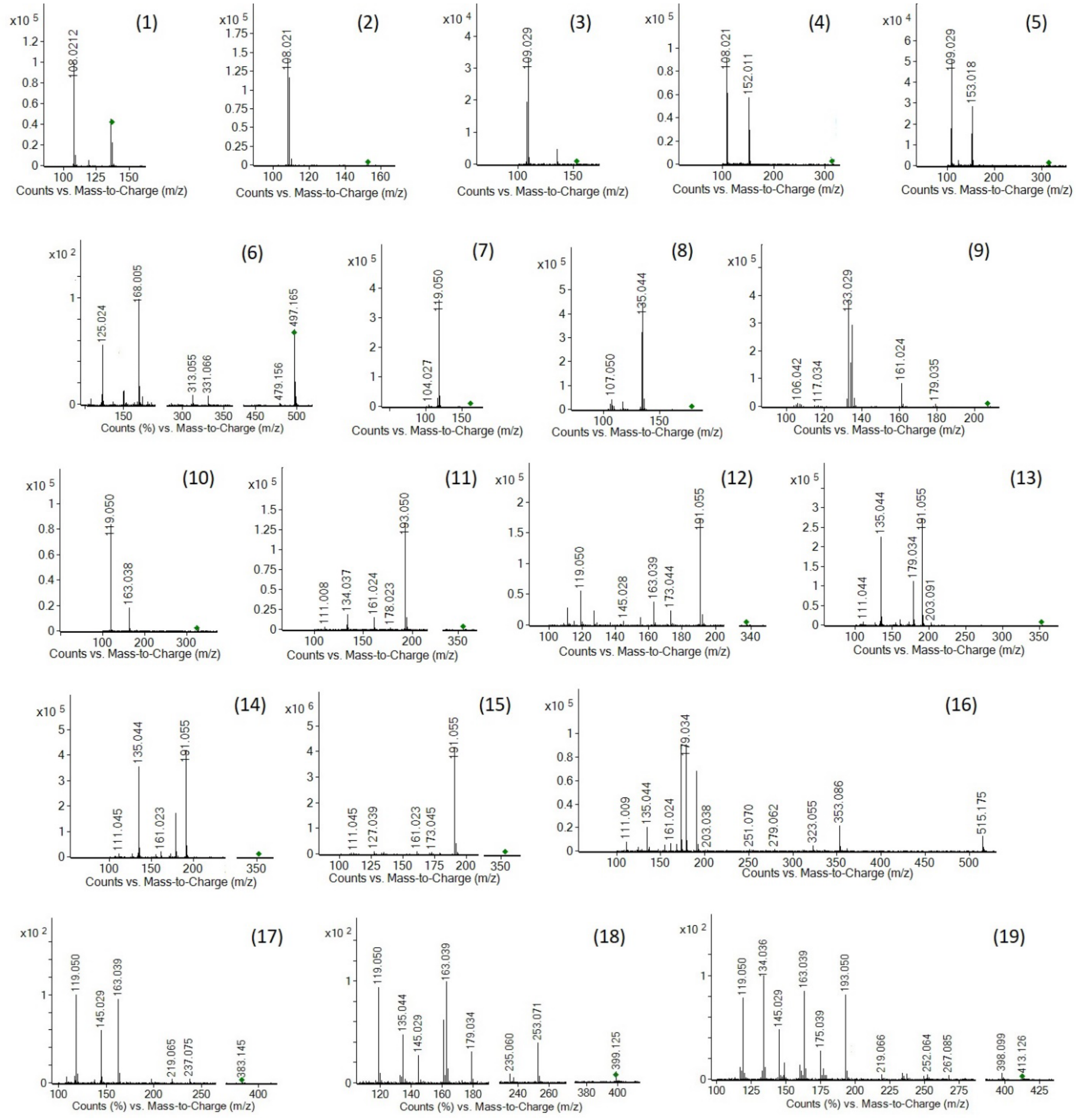

Figure S1. Fragmentation patterns (MS/MS spectra) of identified phenolic acids and derivatives: (**1**) Hydroxybenzoic acid; (**2**) Dihydroxybenzoic acid is. I (Protocatehuic acid); (**3**) Dihydroxybenzoic acid is. II (Gentisic acid); (**4**) Dihydroxybenzoic acid hexoside is. I; (**5**) Dihydroxybenzoic acid hexoside is. II; (**6**) Globulsin B; (**7**) Coumaric acid; (**8**) Caffeic acid; (**9**) Ethyl caffeate; (**10**) Coumaric acid hexoside; (**11**) Ferulic acid hexoside; (**12**) Coumaroylquinic acid; (**13**) Caffeoylquinic acid is. I; (**14**) Caffeoylquinic acid is. II; (**15**) Caffeoylquinic acid is. III (Chlorogenic acid); (**16**) Dicaffeoylquinic acid; (**17**) 1,3-*O*-Dicoumaroyl-glycerol; (**18**) 1,3-*O*-Coumaroyl-caffeoyl-glycerol; (**19**) 1,3-*O*-Coumaroyl-feruloyl-glycerol.

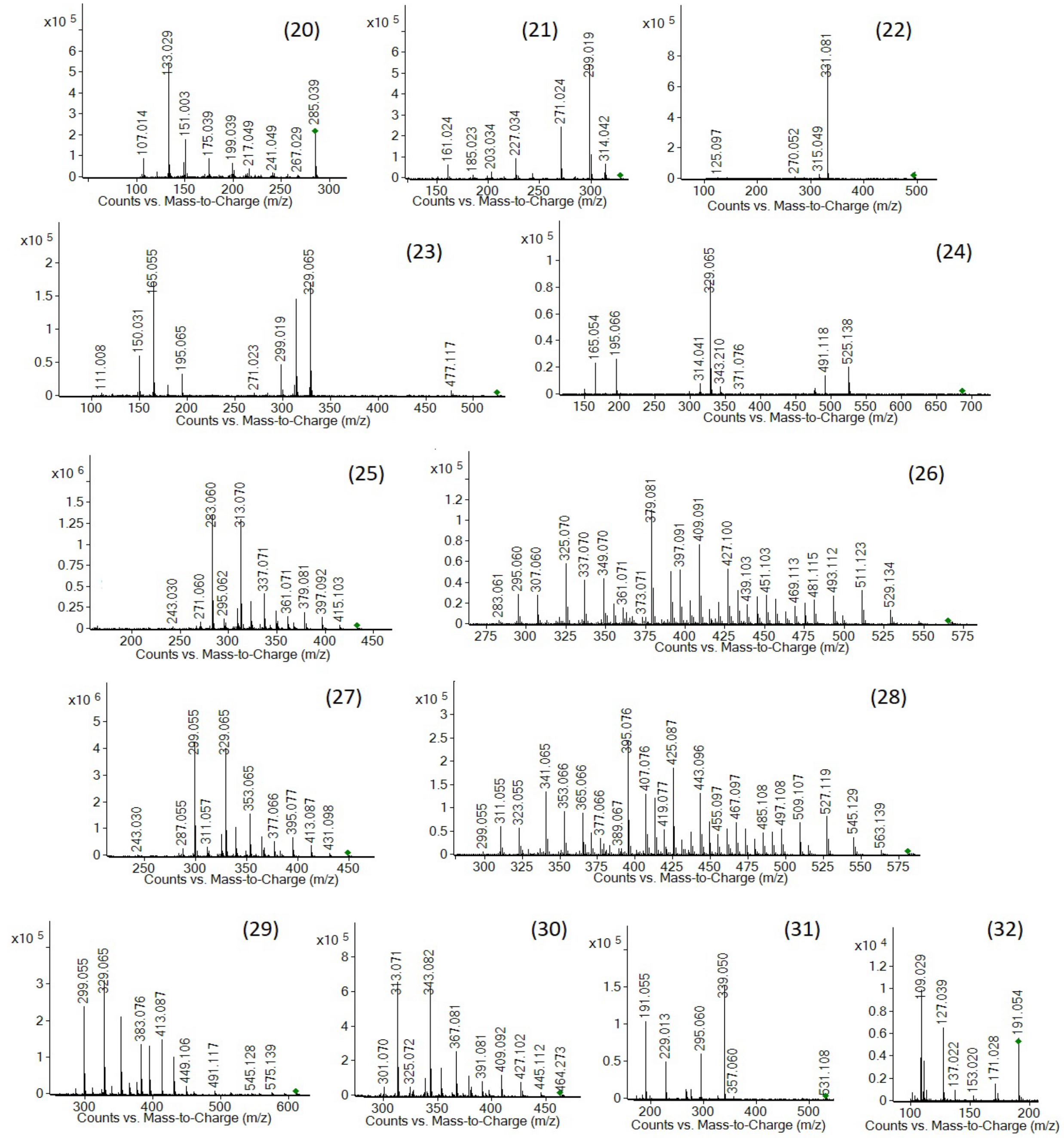

Figure S2. Fragmentation patterns (MS/MS spectra) of identified flavonoids and derivatives, and quinic acid: (**20**) Luteolin; (**21**) Tricin; (**22**) Tricin 7-*O*-hexoside or Tricin 5-*O*-hexoside*; (**23**) Tricin 4′-*O*-(erythro-β-guaiacyl-glyceryl)ether; (**24**) Tricin 4′-*O*-(erythro-β-guaiacyl-glyceryl) ether-7-*O*-hexoside; (**25**) Isovitexin (Apigenin-6-C-glucoside)*; (**26**) Isoschaftoside or Schaftoside (Apigenin-6,8-C-pentoside hexoside)*; (**27**) Isoorientin (Luteolin-6-C-glucoside)*; (**28**) Isocarlinoside or Carlinoside (Luteolin-6,8-C-pentoside hexoside)*; (**29**) Isoorientin 4'-O-glucoside*; (**30**) Isoscoparin (Chrysoeriol-6-C-glucoside)*; (**31**) Acacetin-6-C-(6''-O-malonyl)glucoside; (**32**) Quinic acid. *Compounds (MS/MS spectra) recorded in positive ionization mode.

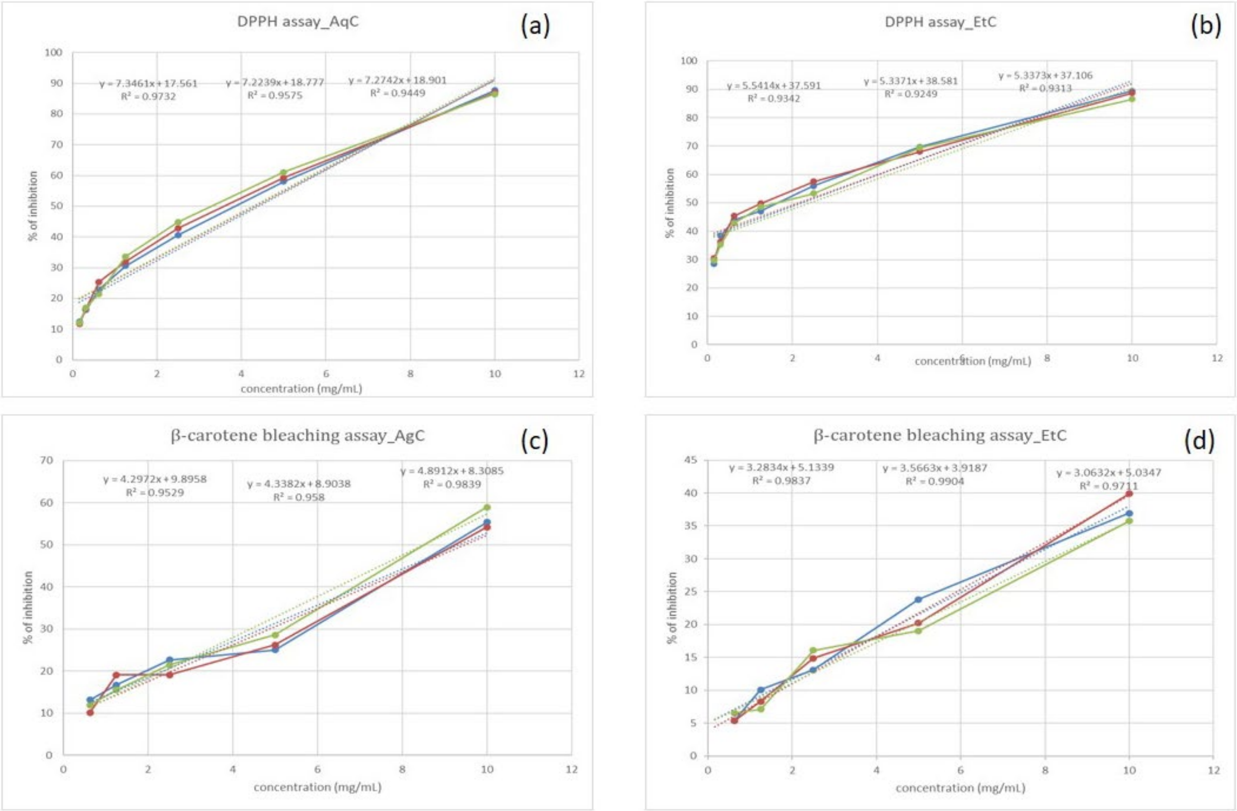

Figure S3. The DPPH (**a**,**b**) and β-caroten bleaching activity (**c**,**d**) of the control extracts at different concentrations (**a**,**c**—AqC, **b**,**d**—EtC). Note: The *x*-axis shows the concentration of sample (mg/mL) and the *y*-axis shows the percentage value of inhibition (%).

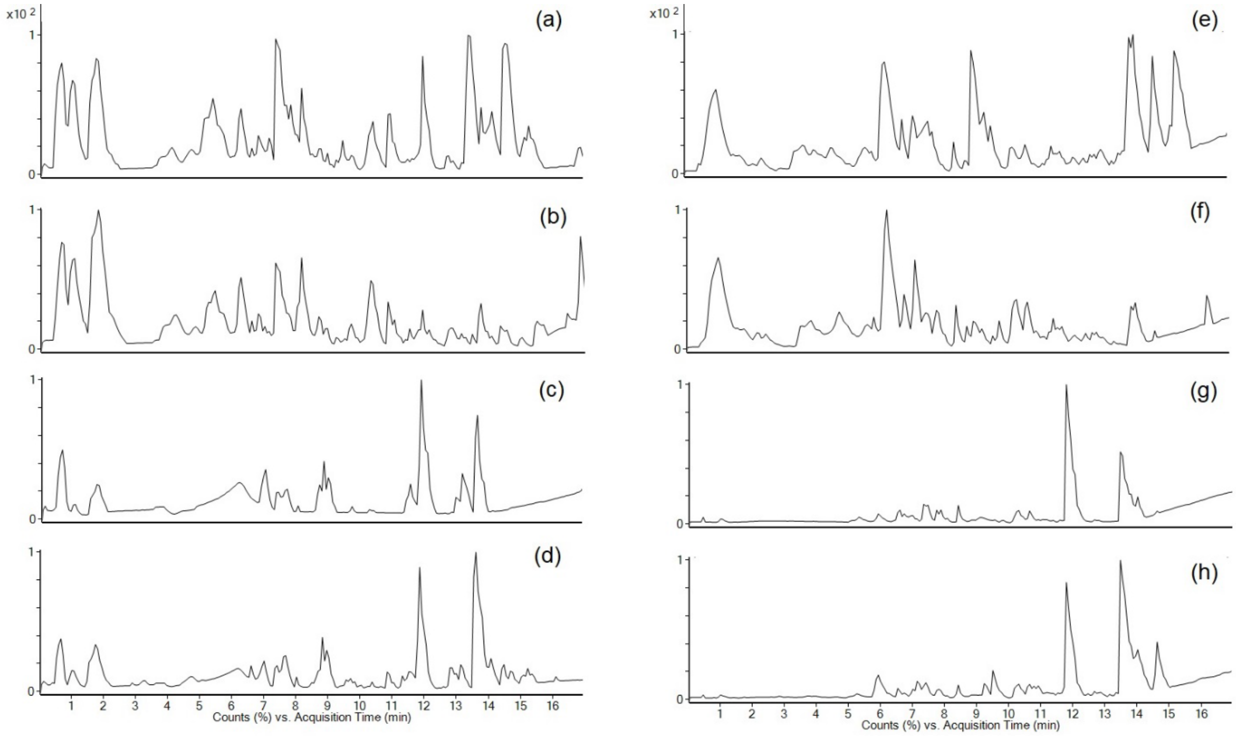

Figure S4. MS-Base peak chromatograms of: (**a**,**e**) Undigested (control) ethanolic extract (EtC); (**b**,**f**) Undigested (control) aqueous extract (AqC); (**c**,**g**) Crude digested aqueous extract (AqD); (**d**,**h**) Crude digested ethanolic extract (EtD); in positive ionisation mode (**a–d**) and negative ionisation mode (**e–h**); for peak annotation see retention time in Table 2.

Table S1. Emax with 95% CI for the highest tested concentration of extracts that produced measurable activity on colorectal cancer cells, and apropriate CI for these extracts.

Extracts	Cell line	Emax with 95% CI	SI
**EtC (24 h)**	HCT-116	88.41 (84.22-92.60)	1.16
	HaCat	103.13 (96.368-109.89)	
**AqC (24 h)**	HCT-116	76.72 (71.92-81.52)	1.39
	HaCat	106.87 (99.46-114.28)	
**AqC (72 h)**	HCT-116	81.61 (76.98-86.23)	1.30
	HaCat	106.41 (104.43-108.39)	
**AqD (24 h)**	HCT-116	83.53 (76.19-90.86)	1.35
	HaCat	113.33 (103.26-123.40)	
